# Cross-cohort microbiome-wide study reveals consistent alterations in the gut bacteriome, but not the gut mycobiome, in patients with hypertension

**DOI:** 10.1128/msystems.00657-25

**Published:** 2025-08-15

**Authors:** Yidan Gao, Dangrang Wang, Tong Lu, Kan Liu, Shenghui Li, Jian Kang, Shanshan Sha, Guorui Xing, Lin Cheng, Shao Fan, Wei Yang, Qiulong Yan, Yanchun Ding, Dafeng Xu

**Affiliations:** 1 Cardiology II Department, The Second Hospital of Dalian Medical University 540418, Dalian, China; 2 Health Management Center, The Second Hospital of Dalian Medical University 540418, Dalian, China; 3 Department of Gastroenterology, The Second Hospital of Dalian Medical University 540418, Dalian, China; 4 The Fifth Affiliated Hospital of Southern Medical University 604348, Guangzhou, China; 5 School of Accounting, Guangzhou Xinhua University 517769, Guangzhou, China; 6 Puensum Genetech Institute, Wuhan, China; 7 Department of Microbiology, College of Basic Sciences, Dalian Medical University 731355, Dalian, China; 8 Department of Laboratory Diagnostics, First Affiliated Hospital of Harbin Medical University 74559, Harbin, China; 9 Department of Nutrition, Chenzhou First People’s Hospital, Chenzhou, China; Drexel University, Philadelphia, Pennsylvania, USA

**Keywords:** hypertension, gut microbiota, metagenomic sequencing

## Abstract

Hypertension, one of the most prevalent cardiovascular diseases, has been linked to the gut microbiota. However, there is a lack of well-defined, cross-population validated gut microbial signatures associated with hypertension, particularly at both the bacterial and fungal levels. To address this gap, we conducted a metagenome-wide analysis of fecal samples from 159 hypertensive patients and 101 healthy controls, using two publicly available data sets from the Beijing and Dalian regions. Our results showed that hypertensive patients exhibit a significant reduction in gut bacterial diversity, accompanied by substantial alterations in bacterial composition. A total of 61 bacterial species were identified with significantly different relative abundance between patients and controls across both regions (combined *P* < 0.05, *q* = 0.25). Hypertension-enriched species included *Lachnospiraceae* (*Clostridium symbiosum*, *Enterocloster bolteae*) and *Clostridium* sp. AT4, while *Lachnospiraceae bacterium*, *Firmicutes bacterium*, and *Clostridium* sp. AM49 4BH were significantly decreased in hypertensive patients. In contrast, no significant differences were observed in gut fungal diversity between hypertensive patients and healthy controls, and only minor differences in fungal composition were noted. Specifically, six fungal species were identified as potentially significant in the combined data set (*P* < 0.05, *q* = 0.73), but they only *Blastomyces emzantsi* c231 meet the same consistency across the two cohorts as the bacterial signatures. Furthermore, we developed classification models using gut bacterial and fungal signatures to distinguish hypertension patients from healthy controls. The bacterium-based models achieved area under the curves (AUCs) greater than 0.70 in cross-cohort classification and validation, while the fungus-based models only achieved AUCs between 0.55 and 0.57. In summary, our study identifies cross-cohort gut bacterial and fungal signatures associated with hypertension, suggesting that the gut bacteriome may serve as a more reliable target for hypertension intervention compared to the gut mycobiome.

**IMPORTANCE:**

Hypertension (HTN) represents a global health burden affecting billions of individuals worldwide; however, the relationship between HTN and gut microbial ecosystems remains inadequately characterized. This study presents the first cross-cohort microbiome analysis revealing significant alterations in the gut bacteriome of HTN patients, with limited changes observed in the mycobiome. These findings highlight the critical role of the gut bacteriome in the pathogenesis of HTN and provide new microbial biomarkers for early diagnosis. Furthermore, the identification of bacterial species establishes a foundation for future intervention approaches, enhancing the applicability of microbiome research in cardiovascular health and opening new avenues for related studies in this field.

## INTRODUCTION

Hypertension (HTN) is a prevalent disease affecting billions of people worldwide. Estimates suggest that by 2025, there will be 1.56 billion adults with HTN, representing 29.2% of the global population (1, 2). It can lead to various cardiovascular events, including myocardial infarction, angina pectoris, and myocardial hypertrophy (3). Additionally, lifestyle factors such as smoking, alcohol consumption, and high-salt diets are linked to the development of HTN (4). Metabolomic analyzes have revealed novel metabolic pathways and disease biomarkers involved in the pathogenesis of HTN (5). Currently, researchers widely acknowledge that the development of HTN is associated with inflammatory responses, immune reactions, and oxidative stress (6, 7). However, due to the complexity and heterogeneity of the condition, the identification of specific causes of HTN remains a significant challenge.

Microorganisms living in the gut, including bacteria, fungi, and viruses, are known as the intestinal microbiota and serve as an indispensable pillar in the sustenance of human health (8). Fecal transfer experiments and gut microbiota remodeling studies demonstrated the critical role of gut microbiota in the pathogenesis of various diseases, including obesity (9), depression (10), chronic intestinal inflammation (11), liver diseases (12), and atherosclerosis (13). Research over the past few decades has shown a relationship between HTN and dysbiosis of the gut microbiota (14). Some studies indicate that the composition of the gut bacteriome and its metabolites including short-chain fatty acids (SCFAs), lipopolysaccharides (LPS), and oxidized trimethylamine N-oxide (TMAO) (15, 16) affect the progression of cardiovascular diseases. Although a direct link between HTN and TMAO has not yet been established, TMAO has been shown to cause arterioles to constrict and increase blood pressure levels when administered to Angiotensin II induced HTN mice (17). Moreover, circulating bacterial wall components such as LPS derived from *Ruminococcus gnavus* and *Coprococcus bolteae* can activate vascular toll-like receptors contributing to low-level chronic inflammation that exacerbates HTN (18). A growing body of evidence indicates that bacteria may contribute to the pathogenesis of HTN in patients.

In addition to bacteria, the gut is also a major reservoir of fungi in the human body. Recent studies indicate that zymosan, a polysaccharide from the *Saccharomyces cerevisiae* cell wall, serves as an inflammatory agent that activates the immune system via inflammatory signaling pathways (19). This activation leads to the release of harmful substances, such as pattern recognition receptors, reactive oxygen species, and cytokines, which can contribute to cardiovascular inflammation and HTN (20). Moreover, some fungal polysaccharides can prevent chronic diseases by mitigating oxidative stress and chronic inflammation (21). For example, mushroom polysaccharides, a type of fungal extract, can activate the immune system, enhancing the body’s resistance to infections. Fungal fiber polysaccharides, such as chitin and chitosan, provide cardiovascular benefits by lowering cholesterol levels and supporting heart health.

These insights not only highlight the crucial role of the gut microbiome in the etiology of HTN but also suggest its potential as a target for interventions aimed at modulating inflammatory and immune pathways. Historically, research on the microbiome was limited due to methodological flaws and insufficient species representation in databases. However, advancements in metagenomic sequencing technology now enable researchers to conduct more detailed and comprehensive studies of gut microbial communities. Given the importance of gut microbes in relation to HTN, we conducted a reanalysis of publicly available metagenomic data sets from two cohorts (15, 16) with totaling 255 human fecal samples. We compared microbial compositions between HTN patients and healthy controls (HCs) using shotgun metagenome-based sequencing data while examining the complex interactions between gut bacteriome and mycobiome. This endeavor aims to uncover previously unexplored mechanisms underlying HTN pathogenesis and to identify viable targets for diagnosis or therapeutic intervention.

## MATERIALS AND METHODS

### Sample information

We reanalyzed 260 fecal metagenomic sequencing samples from two public cohorts to assess the gut microbiota composition between the hypertensive (HTN) group and the HC group. The first cohort (cohort 1) comprised 120 volunteers from Dalian (15), while the second cohort (cohort 2) included 140 volunteers from Beijing (16). All participants reported no antibiotic use in the past 3 months and no significant gastrointestinal disorders, with additional metadata summarized in Yan et al.’s and Li et al.’s studies.

### Construction of gut fungal genome catalog

To create a reliable and high-quality catalog of fungal genomes linked to various human body sites, we conducted an extensive search in the NCBI RefSeq genome database, which contained approximately 6,000 fungal genomes up to November 2024. We manually selected candidate genomes based on their metadata in the BioSample or original studies. The criteria for extraction included: (i) genome size <100 Mb and *N*_50_ length >20 kb, (ii) documentation of the species involved in colonizing or infecting specific human body sites, and (iii) exclusion of diet-derived fungi such as *Agaricus bisporus*, *Auricularia auricula-judae*, and *Ganoderma lucidum*. Ultimately, 1,503 human-associated genomes were retained and grouped into 106 non-redundant species-level clusters using dRep v3.4.0 with the parameters “dereplicate -pa 0.9 -sa 0.96 -nc 0.3S_algorithm fastANI.” The genome with the longest *N*_50_ length for each species was designated as the reference genome. In conclusion, the genomes of these 106 species were used to develop our catalog of gut fungal references.

### Processing of metagenomic sequencing data

To ensure data quality, we first conducted quality control on each sample using fastp v0.20.164 (22). Raw reads underwent multiple filtering steps, including trimming of polyG tails and removing low-quality reads. The specific steps are as follows: (i) removal of reads shorter than 90 bp; (ii) removal of reads with an average Phred quality score below 20; (iii) removal of reads with an average complexity below 30%; (iv) removal of reads where over 30% of their bases have a Phred quality score below 20; and (v) removal of unpaired reads. To minimize the host’s impact on the microbial genome in subsequent analyzes, we mapped the quality-filtered reads to three databases: the MetaPhlAn4 database (23), the chm13 V2.0 genome, and the SILVA rRNA database (24). This step enabled us to exclude reads originating from human sources (25).

For fungal composition analysis, the remaining reads from each sample were aligned against a custom catalog of gut mycobiome genomes using Bowtie2 (26), with end-to-end global alignment (--end-to-end) in fast mode (--fast). To ensure precision, we retained only reads meeting ≥ 95% sequence similarity threshold, applied length-normalized abundance estimation to correct for gene length biases, and required unique or best-quality alignments (MAPQ ≥ 30) for final profiling. Species-level abundances were quantified based on these filtered read counts. For bacterial profiling, high-quality metagenomic reads were aligned to 4,644 reference prokaryotic genomes from the Unified Human Gastrointestinal Genome (UHGG) database (27) using Bowtie2, applying a stringent 95% nucleotide similarity threshold to ensure taxonomic precision. To eliminate potential batch effects arising from different sequencing platforms, we performed batch effect correction using the MMUPHin pipeline (28). Subsequently, the normalized read count was divided by the total of all normalized read counts within the sample, thereby defining the relative abundance of each population. For different microbial taxa, the relative abundance of a taxon was calculated by summing the relative abundances of all populations categorized within that taxon.

### Statistical analyses and visualization

Statistical analyzes were conducted using the R 4.1.2 platform (29). Data visualization was performed using the function “*ggplot*” in the package *ggplot2*, along with the ImageGP 2 and EasyAmplicon platforms (30, 31).

#### Alpha diversity

Species richness was estimated by counting the number of species with relative abundances greater than zero in each sample. We calculate the Shannon’s index and Simpson’s index using the function “*diversity*” from the *vegan* package (32). A high diversity indicates a high richness of species within the sample.

#### Multivariate analyses

Principal coordinate analysis (PCoA) was implemented based on the Bray–Curtis distance at the species levels, using the *‘pcoa’* function from the *ape* package. Permutational multivariate analysis of variance (PERMANOVA) was performed by the “*adonis2*” function from the *vegan* package.

#### Identification of bacterial and fungal markers

We employed MaAsLin2 (33) analysis to identify disease-associated differential signatures, accounting for age, sex, and body mass index (BMI) (considering only data sets with available metadata) as confounding factors. For differential analyzes, the *P* values were calculated using the Wilcoxon rank-sum test or Fisher’s exact test (34), depending on the specific scenario. The combined *P* value of two independent cohorts was calculated based on Fisher’s method. Utilize the following two methods to identify HTN-associated signatures from two cohorts: (i) “combined *P* < 0.05,” consistent enrichment directions and statistical significance (*P* < 0.05) in both cohorts. (ii) The “overall” analysis represents results from MaAsLin2 analysis performed on the merged data set merging both cohorts. The *q* value was used to evaluate the false discovery rate for the correction of multiple comparisons and was calculated based on the R *fdrtool* package.

#### Comparative analysis of gut microbiome co-occurrence networks

To investigate the differences in interaction patterns between fungi and bacteria in the gut of the HC group and HTN patients, we used the platform available at https://www.bioincloud.tech (35) to construct a co-occurrence network. We calculated the correlation between differential bacteria and fungi using Spearman’s rank. Only the inter-microbe correlation coefficients > 0.3 (positive correlation) or <−0.3 (negative correlation) were regarded as strong correlations and included for analysis. The correlation network was visualized using Cytoscape v.3.10.3 software (36).

#### Classification models

We constructed an intra-cohort predictive model based on differential bacterial and fungal signatures associated with HTN, using the random forest function (with *ntree* = 999 and *seed* = 123 for reproducibility) and through a 10-fold cross-validation process repeated 10 times. The model’s effectiveness was evaluated using the area under the receiver operating characteristic curve (AUC), calculated via the “*roc*” function in the *pROC* package. Additionally, the model’s robustness was assessed by training (using random forest with *ntree* = 999 and *seed* = 123) on one cohort and testing on another. The ranking of the significance of bacterial and fungal markers was determined through the importance function.

## RESULTS

### Study population and metagenomic data

To generate gut microbiome profiles, we first filtered raw metagenomic samples to remove low-quality reads resulting in a total of approximately 1.5 Tbp of clean reads. Demographic analysis indicated no significant differences in gender, age, or BMI between HC and HTN patients in cohort 1 and cohort 2 (Table S1). Additional subject metadata is summarized in the studies by Li et al.’s and Yan et al.’s (15, 16).

### Altered gut bacterial structure in HTN patients

**Fig 1:**
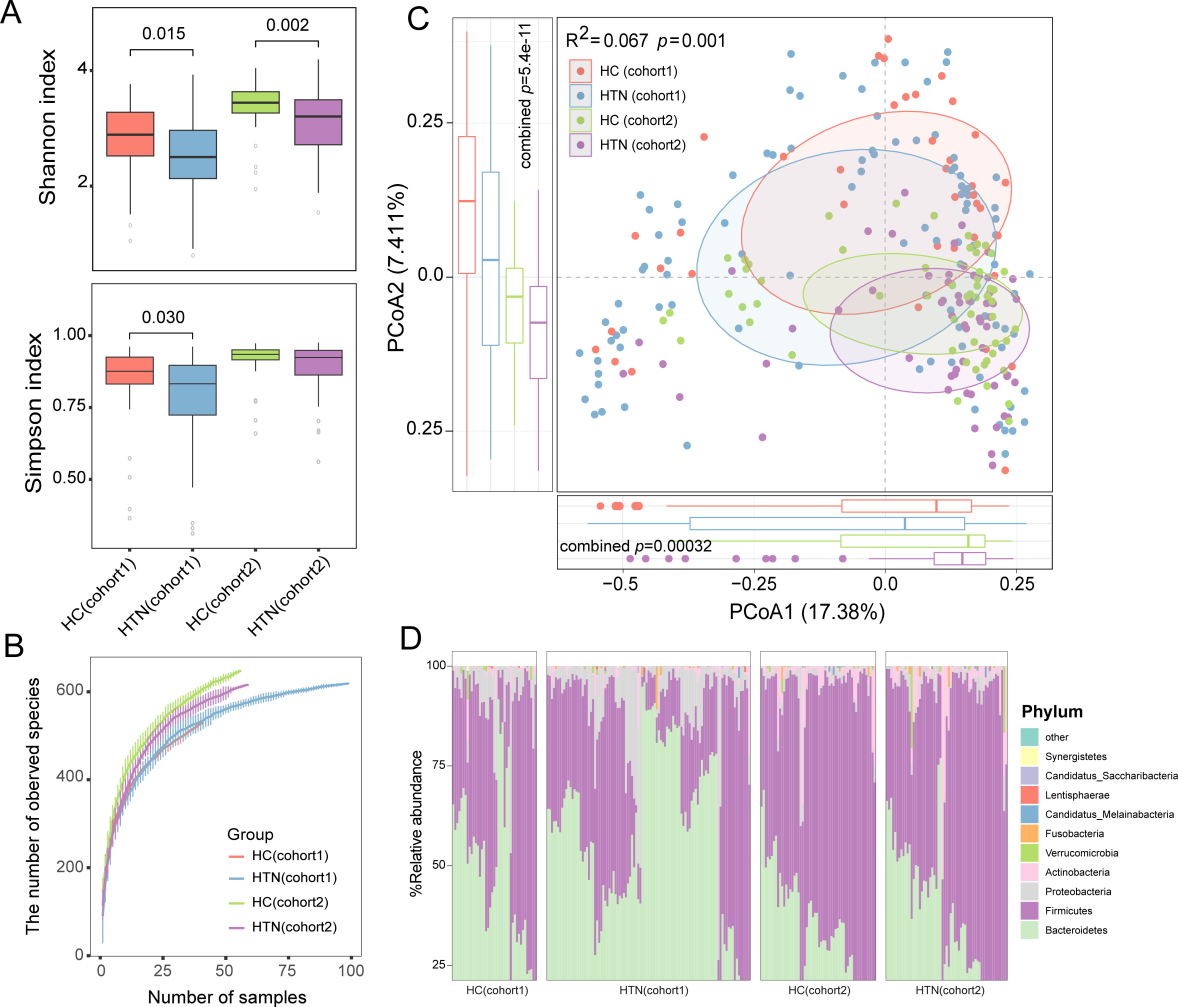
Comparison of gut bacteriome diversity and composition between HTN patients and healthy controls. (**A**) Shannon index (upper), Simpson index (bottom). (**B**) Rarefaction curve analysis of the number of observed bacterial (**C**) PCoA using the Bray-Curtis distance of the bacterial-level profiles. Samples are plotted on the top two principal coordinate axes (PCoA1 and PCoA2), along with the corresponding ratio of variance explained by these axes. Ellipsoids surrounding each group indicate a 95% confidence interval. The bottom and left box plots summarize the distribution of PCoA1 and PCoA2 values for each group. The texts above the PCoA figure indicate the effect size (*R*^2^) of HTN species on the gut bacteriome, as determined using the Adonis test. (**D**) Distribution of the top 11 abundant species across all samples.

We first analyzed the gut bacterial composition of all fecal samples using the available bacterial genome database in the UHGG, obtaining the gut prokaryotic profile, referred to as the “gut bacteriome.” This profile included a total of 1,432 bacterial and archaeal taxa encompassing 12 phyla, 59 classes, 76 orders, 101 families, 366 genera, and 730 species. To evaluate the alpha diversity of the gut bacteriome in both cohorts, we calculated the number of observed bacterial taxa and the diversity index for each sample, comparing these indices between HTN and HC groups (Fig. 1A). Rarefaction analysis indicated no significant difference in the number of observed bacterial species between the HTN and HC groups across the two cohorts (Fig. 1B). However, HTN patients exhibited significantly reduced Shannon and Simpson diversity indices in both cohorts (Wilcoxon rank-sum test, *P* < 0.05). To further investigate the differences in gut bacteria between groups from different cohorts, we conducted PCoA and PERMANOVA analyzes. The PCoA based on Bray-Curtis distances of species-level composition revealed that the top two principal coordinate axes (PCoA1 and PCoA2) explained 17.38% and 7.41% of the total variation, respectively. Furthermore, PERMANOVA indicated a significant difference in gut microbiota between HTN patients and HC from different cohorts (*R*^2^ = 0.067, *P* = 0.001; Fig. 1C), suggesting substantial changes in gut bacteria among hypertensive patients. In addition, we compared the gut bacteriome between the HTN and HC subjects at the species level across two cohorts. *Prevotella copri* clade A was the first most abundant species, while other common genera, such as *Faecalibacterium prausnitzii* and *Sutterella wadsworthensis*, had relatively high abundances in both cohorts (Fig. 1D).

### Gut bacterial signatures associated with HTN

**Fig 2:**
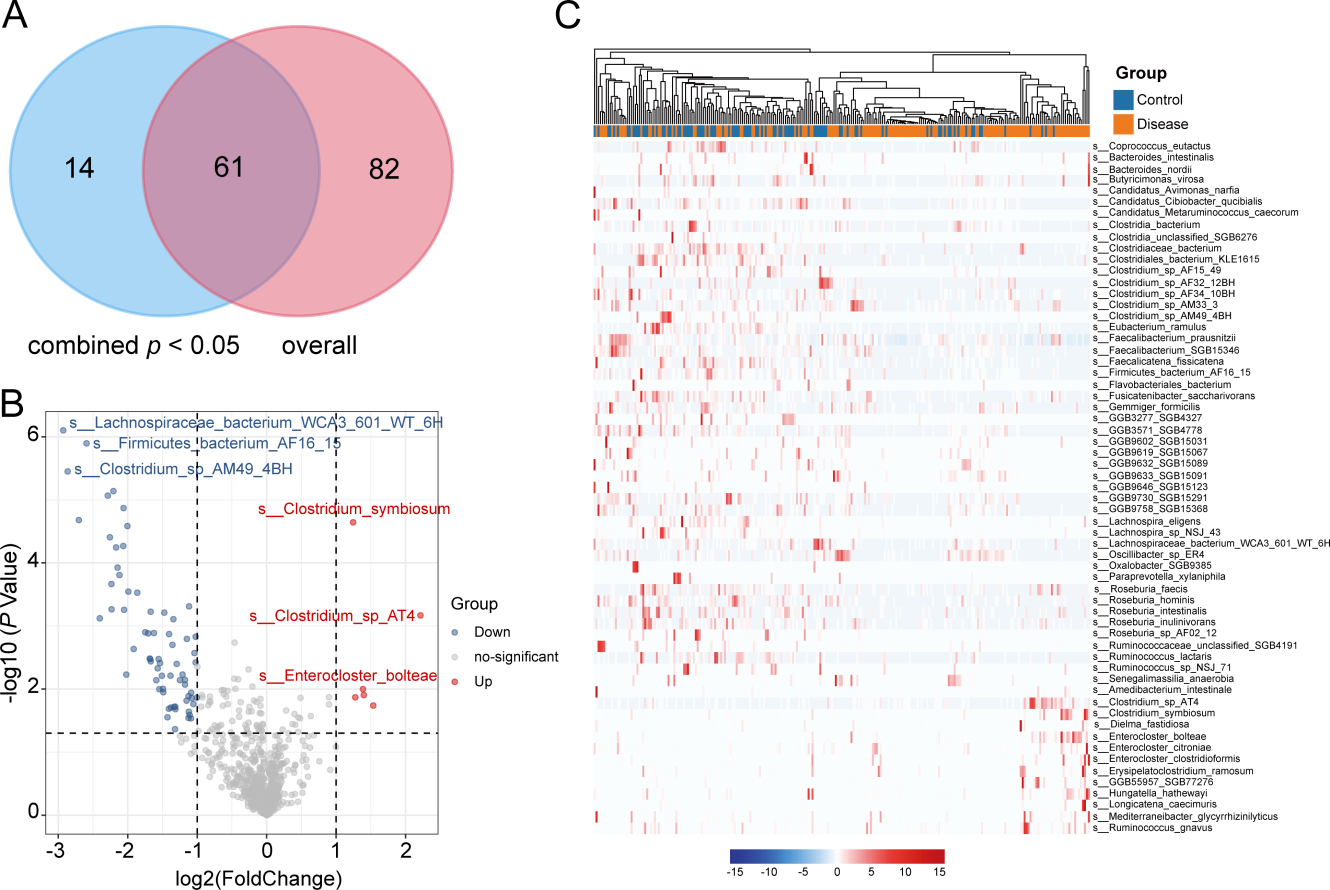
Bacterial signatures associated with HTN. (**A**) Overlap of the differentially identified bacterial taxa from combined *P* and overall. (**B**) Volcano plot shows the fold change versus *P* values for all bacteria. The *x*-axis shows the ratio of bacterial abundance in HTN patients compared with that in healthy controls (HCs). The *y*-axis shows the *P* value (−log10 transformed) of a bacterial. The 61 HTN-associated bacteria that were enriched in HTN and HC subjects are shown with red and blue points, respectively. (**C**) A heatmap showing the proportional contribution of 61 HTN-associated species. The species that were enriched in the gut microbiota of HTN patients and HCs are labeled in red and blue, respectively.

We conducted MaAsLin2 analysis (33) to identify differential bacterial signatures between HTN patients and HC in both cohorts. At the species level, we first analyzed the gut bacteriome of the two regions and calculated their combined *P* values. We identified 75 bacterial taxa that showed consistent enrichment directions and significant differences between the two cohorts (Fisher’s exact test, combined *P* < 0.05). Subsequently, we merged the samples from the two cohorts and identified 143 significantly different bacterial species (Wilcoxon rank-sum test, *P* < 0.05). The intersection of the differentially bacteria from the two methods yielded a total of 61 species with *q* < 0.05 (Fig. 2A; Table S2), which were regarded as reliable HTN-associated signatures. Notably, HTN patients showed a significant reduction in the dominant gut bacterial species such as *Lachnospiraceae bacterium*, *Firmicutes bacterium*, and *Clostridium* sp. AM49 4BH compared to the HC group. Conversely, various opportunistic pathogens including *Clostridium symbiosum*, *Clostridium* sp. AT4, and *Enterocloster bolteae* were enriched in HTN patients (Fig. 2B and C).

### Altered gut fungal structure in HTN patients

**Fig 3:**
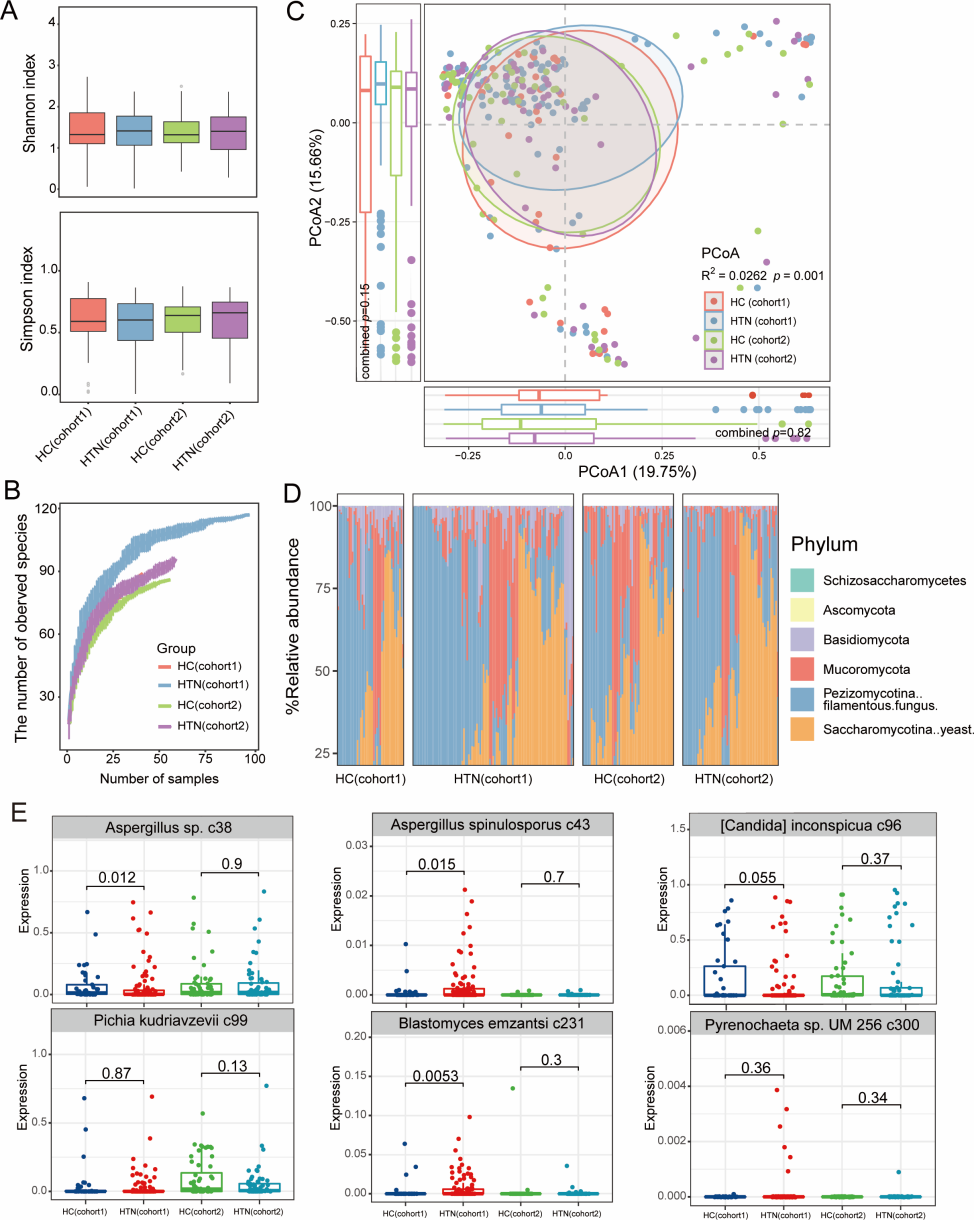
Comparison of gut mycobiome diversity and composition between HTN patients and healthy controls. (**A**) Shannon index (upper), Simpson index (bottom). (**B**) Rarefaction curve analysis of the number of observed fungi (**C**) PCoA using the Bray-Curtis distance of the fungal-level profiles. Samples are plotted on the top two principal coordinate axes (PCoA1 and PCoA2), along with the corresponding ratio of variance explained by these axes. Ellipsoids surrounding each group indicate a 95% confidence interval. The bottom and left box plots summarize the distribution of PCoA1 and PCoA2 values for each group. The texts above the PCoA figure indicate the effect size (*R*^2^) of HTN species on the gut mycobiome, as determined using the Adonis test. (**D**) Distribution of the top 11 abundant species across all samples. (**E**) Fungus signatures associated with HTN.

Next, we mapped the reads from each sample against the fungal genomes from the NCBI RefSeq database to obtain the gut fungal profile referred to as the “gut mycobiome,” which comprised a total of 207 fungal taxa including 3 phyla, 6 classes, 13 orders, 25 families, 42 genera, and 127 species. We then analyzed the alpha diversity of the gut mycobiome using the same approach as for the gut bacteriome (Fig. 3A). Rarefaction analysis revealed that the number of guts mycobiome taxa in HTN patients was significantly lower than that in the HC group across both cohorts (Fig. 3B). However, there were no significant changes in the Shannon and Simpson diversity indices between the groups in either cohort (Wilcoxon rank-sum test, *P* < 0.05). Similarly, we conducted PCoA and PERMANOVA analyses for the gut mycobiome. PCoA indicated that the top two principal coordinate axes accounted for 19.75% and 15.66% of the variation, respectively. Furthermore, PERMANOVA revealed a significant effect of HTN species on the composition and structure of the gut mycobiome in both cohorts (*R*^2^ = 0.0262, *P* = 0.002; Fig. 3C). At the species level, *Aureobasidium* was the first most abundant fungal genus, while other common species, such as *Rhizopus stolonifer* and *[Candida] inconspicua*, had relatively high abundances in both regions (Fig. 3D).

Similarly, we used MaAsLin2 analysis to identify differential fungal signatures between HTN patients and HC subjects at the species level. When we analyzed the gut mycobiome of the two cohorts and calculated their combined *P* values, only *Blastomyces emzantsi* c231 showed consistent enrichment directions between the two cohorts (Fisher’s exact test, combined *P* < 0.05). Upon merging the two cohorts, we identified only six differentially abundant fungi (Wilcoxon rank-sum test, *P* < 0.05; Fig. 3E; Table S2). Across two cohorts, *Aspergillus spinulosporus* c43 (merged *P* = 0.0008), *B. emzantsi* c231 (merged *P* = 0.0009), and *Pyrenochaeta* sp. UM 256 c300 (merged *P* = 0.048) were slightly enriched in HTN patients compared to HCs, whereas *Aspergillus* sp. c38 (merged *P* = 0.003), *[Candida] inconspicua* c96 (merged *P* = 0.014), and *Pichia kudriavzevii* c99 (merged *P* = 0.027) were slightly depleted in patients.

### Comparative analysis of gut microbiome co-occurrence networks

**Fig 4:**
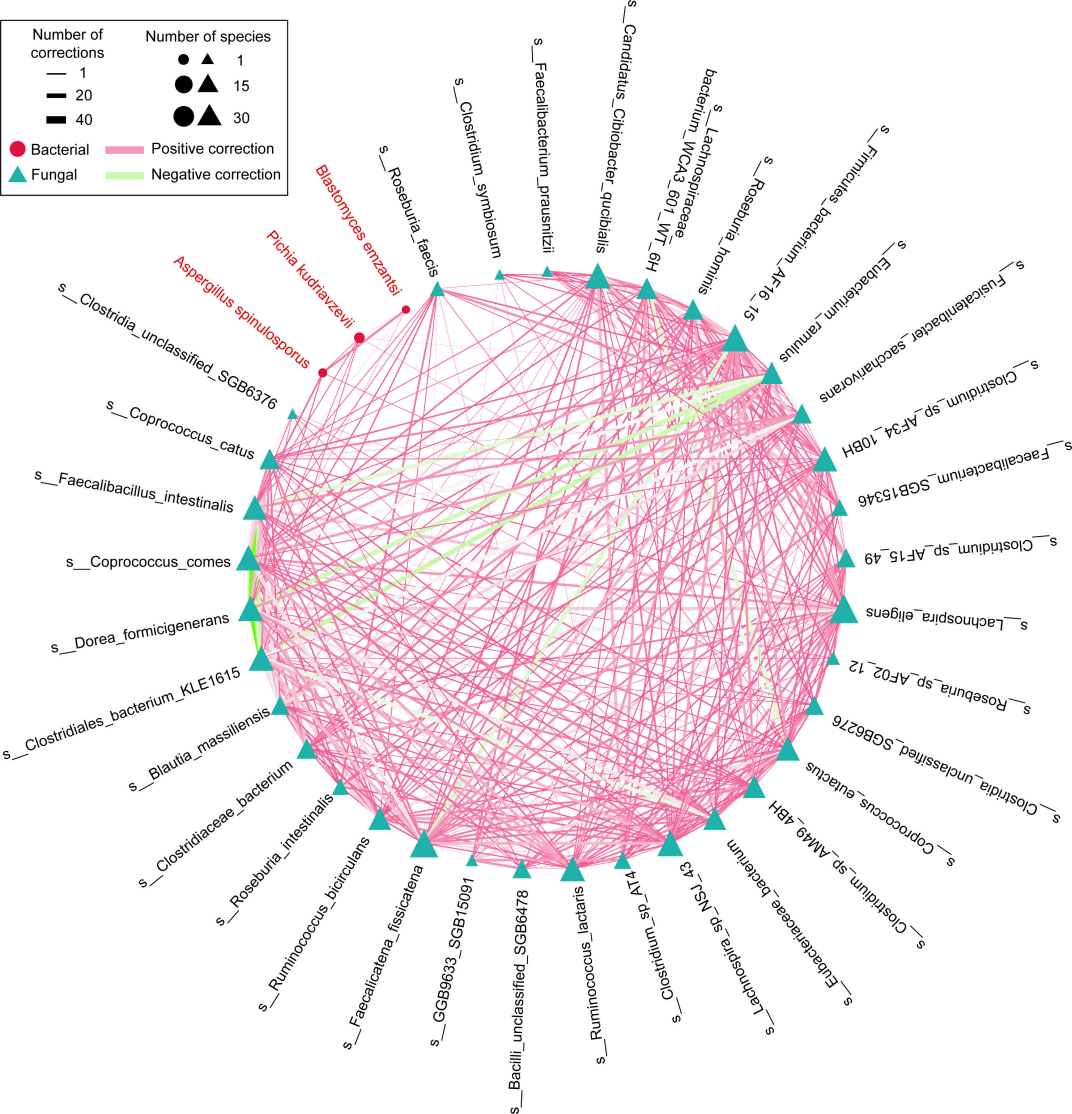
Correlation analysis among gut bacteriome and mycobiome. The network illustrates the correlations between differentially abundant gut bacteriome and mycobiome in HTN patients and the HC group.

Given the coexistence of various microorganisms, including bacteria and fungi, in the gut that actively collaborate to form a gut microbiome network, further research on the interactions between the gut microbiome and the fungal community is crucial. We constructed gut microbial networks using fungal and bacterial species that exhibited significant differential abundance between HTN patients and HC group (Fig. 4). Although there is a positive correlation between gut bacteriome and mycobiome, this correlation is quite small. Most interactions among bacteria in the network are characterized by positive correlations. Notably, *Eubacterium ramulus* exhibits a significant negative correlation with several bacteria, including *Lachnospiraceae* (*Dorea formicigenerans, Faecalicatena fissicatena*) and *Clostridiales bacterium* KLE1615. These bacteria may play central roles in the network. These findings provide valuable insights into the specific microbial structure within the gut microenvironment related to HTN.

### Diagnostic potential of gut microbial biomarkers

**Fig 5:**
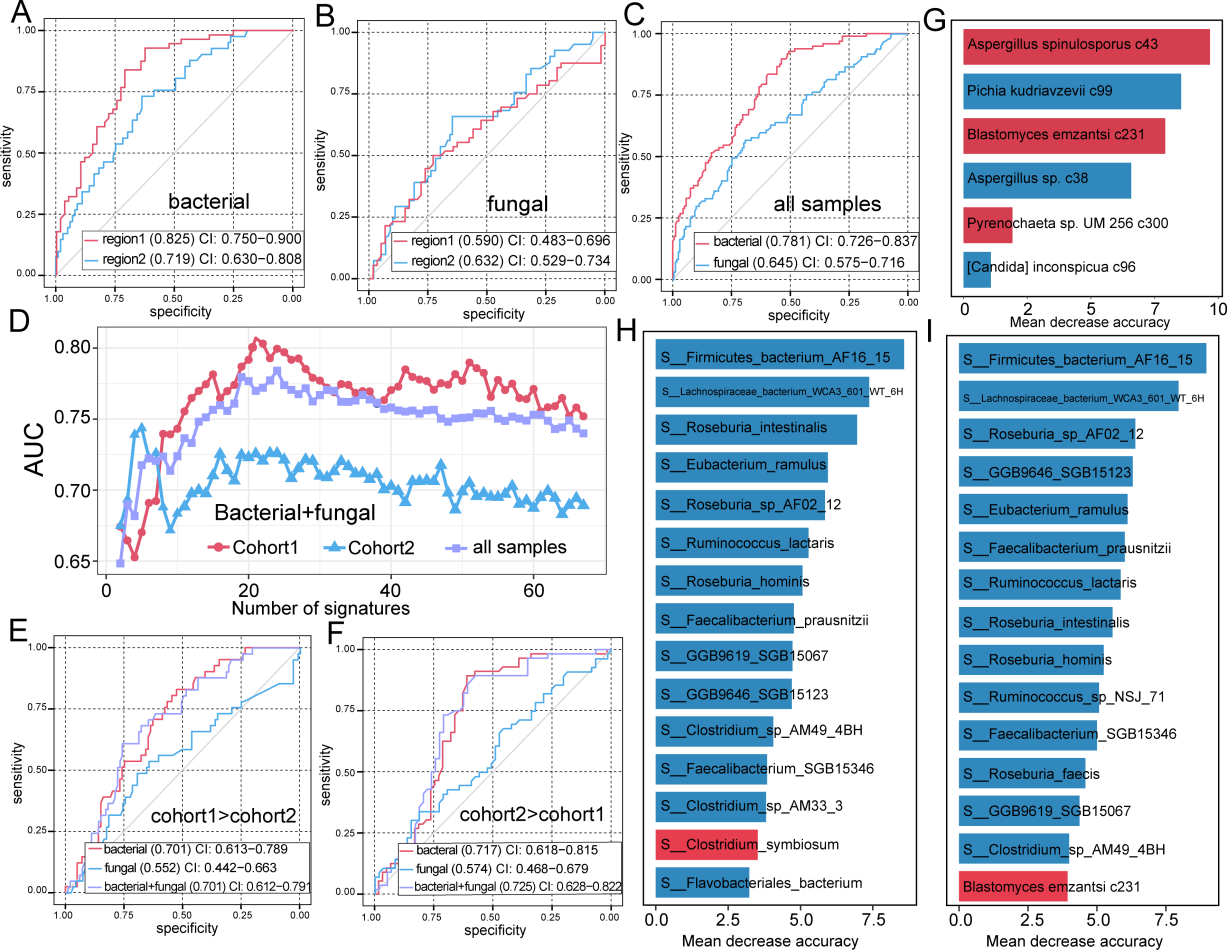
The performance of the random forest model for classifying HTN species. (**A**) Receiver operating characteristic (ROC) curve analysis showing the performance of the random forest model for classifying HTN species based on all HTN-associated bacteria. (**B**) ROC curve analysis showing the performance of the random forest model for classifying HTN species based on all HTN-associated fungi. (**C**) ROC curve analysis showing the performance of the random forest model for classifying HTN species based on all samples. (**D**) The line chart showing average AUC values of the random forest models using different numbers of the top important HTN-associated bacteria and fungi. (**E**) ROC curve analysis showing the performance of the cross-cohort models (train on cohort 1 and classify on cohort 2). (**F**) ROC curve analysis showing the performance of the cross-cohort models (train on cohort 2 and classify on cohort 1). (**G**) The six most important fungal signatures. (**H**) The 10 most important bacterial signatures. (**I**) The 15 most important bacterial and fungal signatures.

To investigate the potential of gut bacterial and fungal signatures for classifying HTN species, we built a random forest model with 10 iterations of 10-fold cross-validation to obtain the AUC. The bacterium-based models for both cohorts demonstrated acceptable accuracy for discriminating between HC and patients, with AUCs greater than 0.7 (cohort 1: 0.825 and cohort 2: 0.719) (Fig. 5A). In contrast, the fungus-based models only achieved AUCs of approximately 0.6 (cohort 1: 0.590 and cohort 2: 0.632) (Fig. 5B). To address the limitations of sample size in a single cohort, we constructed a random forest model using a combined pool of all samples from both cohorts. The model achieved AUCs of 0.781 for bacterial and 0.645 for fungal (Fig. 5C). Furthermore, we trained new random forest models using different numbers of bacterial and fungal signatures to explore the optimal number of signatures for dividing HTN patients and HC subjects. These models achieved the highest discriminatory powers of the AUCs of 0.807, 0.744, and 0.784 in cohort 1, cohort 2, and across all samples, respectively (Fig. 5D).

Then, we constructed models based on bacterial, fungal, and all differential microbial for cross-cohort predictions. The models trained on cohort 1 achieved AUCs of 0.701, 0.552, and 0.701 for bacterium-based, fungal-based, and microbial-based models, respectively, when classifying patients and controls in cohort 2 (Fig. 5E). Conversely, models trained on cohort 2 achieved AUCs of 0.717, 0.574, and 0.725 for these models for classifying patients and controls in cohort 1 (Fig. 5F). Overall, the bacterium-based model exhibited significantly better predictive performance than the fungus-based model, suggesting that these biomarkers warrant further exploration in disease diagnosis. In the random forest model, several species, such as *Pichia kudriavzevii*, *Aspergillus* sp., and *F. bacterium* AF16 15, enriched in HC, *Aspergillus spinulosporus*,* B. emzantsi*, and *C. symbiosum*, enriched in HTN, exhibited the highest identification importance (Fig. 5G through I).

## DISCUSSION

Due to factors such as aging populations and environmental influences, HTN has emerged as one of the fastest-growing cardiovascular disorders worldwide (37). An increasing number of studies emphasize the role of gut microbiota in the pathogenesis of various cardiovascular diseases (14). Gut microbiota is a key regulator of intestinal barrier function and plays a crucial role in the etiology of HTN. Disruption of the gut microbiota can result in the entry of endotoxins (e.g., LPS) (38) into the host, leading to inflammation and cardiovascular events (39). Currently, various factors have been proposed to explain the microbiome’s role in HTN, including effects on gut barrier integrity, promotion of inflammatory cascades, and influence on endocrine signaling and SCFA production (40). However, these factors do not fully elucidate HTN’s etiology, prompting researchers to explore additional potential causes (41). Thus, we studied the gut bacteriome and mycobiome of two cohorts, consisting of a total of 159 HTN patients and 101 HCs, using shotgun metagenomics.

We observed a noteworthy decrease in bacterial diversity among HTN patients. This is a common characteristic in various cardiovascular diseases (42) indicating the emergence of gut bacteriome dysbiosis. Notably, the disease state contributed to 16.6% of the variance in the gut bacteriome. This proportion is significantly larger than that observed in other cardiovascular conditions such as heart failure (43) and cerebral ischemic stroke (44), suggesting that HTN patients may experience more pronounced shifts in their microbiota. Specifically, we identified an enrichment of *L. bacterium*, *F. bacterium*, and *Clostridium* sp. AM49 4BH in the gut bacteriome of HC. Previous studies have shown that *L. bacterium* is negatively correlated with metabolic diseases, including type 2 diabetes and obesity. It is more abundant in HC individuals compared to those with hyperuricemia, suggesting a protective role in intestinal barrier function. Furthermore, *L. bacterium* has been identified as a key species in obesity resistance, highlighting its potential importance in metabolic health warranting further investigation (45). *F. bacterium* is positively correlated with anandamide (AEA), which is linked to the endogenous cannabinoid system. This relationship suggests that changes in *F. bacterium* may improve anxiety-like behaviors through the modulation of AEA levels (46). Similarly, *Faecalibacillus* is associated with inflammation. Its abundance is linked to elevated blood pressure, suggesting a potential role in HTN regulation (47). Furthermore, the three most significantly enriched fungal in HTN patients are *Lachnospiraceae* (*C. symbiosum, E. bolteae*), and *Clostridium* sp. AT4. Lachnospiraceae overgrowth in the gut is associated with detrimental functions and has been observed in various conditions, including ischemic stroke, diabetes mellitus, and inflammatory bowel disease (48–50). Additionally, *Lachnospiraceae* genera could lead to decreased intestinal butyrate production in hypertensive individuals, which in turn contributes to lower levels of HDL-C via apoA-IV gene regulation, leading to increased blood pressure and promoting HTN (51). Previous studies have shown that Phe-Phe produced by *Clostridium* sp. AT4 is highly abundant in the obesity-depression comorbidity group, suggesting that its biological role in cellular inflammatory processes may underlie this comorbidity (52). In summary, these bacteria may serve as potential indicators of gut health. Our findings could enhance the understanding and interpretation of the disease’s etiology.

The gut fungal community of HTN patients revealed an increased relative abundance of pathogenic *Eurotiomycetes* (*B. emzantsi* c231*, A. spinulosporus* c43) and *Pyrenochaeta* sp. UM 256 c300, along with a decreased abundance of beneficial fungal such as *Aspergillus* sp. c38 and *Pichia* (*[Candida] inconspicua* c96*, P. kudriavzevii* c99). Currently, case reports on *Eurotiomycetes* (*B. emzantsi* c231*, A. spinulosporus* c43) are limited. The few reported cases associated with *B. emzantsi* c231 are primarily from Southern Africa. Similarly, the only report on *A. spinulosporus* c43 involves a 22-month-old boy from South Korea. He was diagnosed with central nervous system aspergillosis and meningitis, from which *A. spinulosporus* was isolated (53, 54). Moreover, *Pyrenochaeta* sp. UM 256 c300 is also a rare human pathogen that causes infections in human skin and nails (55). Based on the aforementioned harmful fungi, it is evident that the types of diseases caused by fungi are relatively limited. Similar issues are observed among these beneficial fungi. Regarding *Pichia* fungal, *[Candida] inconspicua c96* has only been observed in patients with hematological malignancies (56, 57). *P. kudriavzevii* c99, which is found at higher levels in the fecal samples of nonalcoholic steatohepatitis (NASH) patients. It produces ethanol levels ten times greater than those of bacteria while generating high triglyceride levels *in vitro* and converting fructose to ethanol, which could significantly influence NASH progression. *Aspergillus* sp. c38 produces metabolites such as *sterigmatocystins*, which exert anti-neuroinflammatory effects by inhibiting the production of nitric oxide and pro-inflammatory cytokines like TNF-alpha and IL-6 in LPS-stimulated BV2 cells (58). In summary, the differences in gut microbiota between HTN patients and HC may be a contributing factor to HTN.

Research on the interactions among various microorganisms in the gut ecosystem related to HTN is currently limited. Network analysis results indicate that bacterial interactions significantly exceed bacterial-fungal interactions, suggesting that dysregulation in HTN may be related to bacterial interactions. This pattern diverges fundamentally from atherosclerotic cardiovascular disease microbiomes, where fungal-bacterial alliances critically mediate pathogenesis (59). Research indicates that under low dietary fiber conditions, *E. ramulus* may compete with certain members of the *Lachnospiraceae* for the same carbon sources, such as polysaccharides and flavonoids, resulting in a negative correlation in abundance. For instance, in the intestines of patients with irritable bowel syndrome, *E. ramulus* shows a negative correlation with *Lachnospiraceae* abundance (Spearman *r* = −0.42), which may be associated with differences in bile acid metabolism. Additionally, in the high-fat diet group, the abundance of *E. ramulus* increases while that of *Lachnospiraceae* (e.g., Blautia) decreases, suggesting a potential for nutritional competition (60). These results indicate that bacterial interactions play a crucial role in the development of HTN, and targeted therapeutic regulation of key species, such as *E. ramulus,* to restore microbial balance could serve as a potential clinical approach for treating HTN. Future studies should validate these network properties through fecal transplantation experiments and metabolomic profiling of the implicated pathways.

Currently, despite extensive efforts to identify biomarkers for the early detection of HTN, the critical role of the microbiome in screening for HTN biomarkers is often overlooked. We found that the bacterium-based models demonstrated high predictive performance within the intra-cohort evidenced by AUCs greater than 0.70 in both cohorts, while the fungus-based models had AUCs of approximately 0.60. Similarly, during cross-cohort predictions, the predictive performance of the gut bacterium-based models remained superior to that of the fungus-based models. These findings highlight the diagnostic potential of the gut bacteriome, rather than the mycobiome, in distinguishing HTN.

This study has several limitations that should be considered: (i) Sample size and demographic variability: The relatively small cohort size (particularly the HC group, *n* = 101) may reduce statistical power to detect subtle microbial differences. Additionally, while we adjusted for age and sex in our models, residual confounding may exist due to uneven distributions across subgroups. (ii) Correlational design: The cross-sectional nature of our data precludes causal inferences between microbiota changes and HTN onset. (iii) While we identified taxonomically distinct microbial signatures, the absence of multi-omics integration (particularly metabolomics) and experimental validation. We acknowledge this observation cannot exclude potential key roles of low-abundance fungi through highly bioactive metabolites (e.g., immunomodulatory polysaccharides) or bacterial-fungal interactions. Future studies combining metagenomics with metabolomic profiling and *in vitro* coculture systems will be essential to dissect these cross-kingdom functional relationships. (iv) Database constraints: The construction of fungal databases involves a subjective selection process, with no more objective alternatives currently available. In this article, we constructed a fungal genome collection using previous methods (25). Furthermore, dietary fungi may significantly affect the accuracy of fungal composition in some samples, impacting the analysis results. Nevertheless, these findings provide a clinically actionable framework for future research.

### Conclusions

In conclusion, notable differential bacterial and fungal signatures that distinguish HTN patients from HC were identified using cross-cohort metagenomic shotgun sequencing data. Our findings demonstrate that HTN significantly impacts the gut bacteriome, while its effect on the gut mycobiome is minimal. Furthermore, both the intra-cohort and cross-cohort random forests predictive models indicated that bacterium-based models exhibited high efficacy and reproducibility, indicating significant clinical potential, whereas fungus-based models performed less effectively. Therefore, these bacterial signatures show promise as novel targets for HTN intervention, providing new avenues for preventive and therapeutic strategies.

## SUPPLEMENTAL MATERIAL

Table S1 msystems.00657-25-s0001.doc Detailed phenotype information of primary hypertensive patients and matched heathy controls.

Table S2 msystems.00657-25-s0002.doc Detailed information of 61 gut bacterial signatures.

ASM does not own the copyrights to Supplemental Material that may be linked to, or accessed through, an article. The authors have granted ASM a non-exclusive, world-wide license to publish the Supplemental Material files. Please contact the corresponding author directly for reuse.
