# Supplementary material for: Cross-cohort microbiome-wide study reveals consistent alterations in the gut bacteriome, but not the gut mycobiome, in patients with hypertension
Source: mSystems. 2025 Aug 15;10(9):e00657-25. doi: 10.1128/msystems.00657-25 (PMC12455987; doi:10.1128/msystems.00657-25)
Supplement: Table S1 — Detailed phenotype information of primary hypertensive patients and matched heathy controls. [file msystems.00657-25-s0001.doc]

| **Table S1 | Detailed phenotype information of primary hypertensive patients and gender-, age- and BMI-matched heathy controls.** | | | | | | | | | | | | | | | | | |  |
| --- | --- | --- | --- | --- | --- | --- | --- | --- | --- | --- | --- | --- | --- | --- | --- | --- | --- | --- |
| **Sample ID** | **Sex** | **Age (y)** | **Height (cm)** | **Weight (kg)** | **BMI (kg/m2)** | **Current SBP (mmHg)** | **Current DBP (mmHg)** | **HT stage*** | **Disease duration (y)** | **FGB (mmol/L)** | **HDL (mmol/L)** | **LDL (mmol/L)** | **TG (mmol/L)** | **TC (mmol/L)** | **Smoke (Y/N)** | **T2D (Y/D)** | **Group** | **Cohort** |
| D01 | M | 44 | 174 | 78 | 25.8 | 140 | 86 | 1 | 5 | 8.78 | 1.08 | 3.21 | 1.75 | 4.89 | N | Y | Hypertension | Dalian |
| D02 | F | 65 | 170 | 58 | 20.1 | 150 | 90 | 1 | 0 | 7.33 | 1.28 | 3.94 | 2.02 | 4.94 | N | Y | Hypertension | Dalian |
| D03 | F | 59 | 162 | 65 | 24.8 | 159 | 94 | 1 | 0 | 5.81 | 1.28 | 3.74 | 1.36 | 5.78 | N | N | Hypertension | Dalian |
| D04 | F | 58 | 155 | 62 | 25.8 | 200 | 130 | 3 | 5 | 9.8 | 1.12 | 3.79 | 2.73 | 5.53 | Y | Y | Hypertension | Dalian |
| D05 | M | 47 | 169 | 52 | 18.2 | 150 | 92 | 1 | 6 | 5.83 | 1.16 | 2.94 | 2 | 4.11 | N | N | Hypertension | Dalian |
| D06 | M | 52 | 165 | 64 | 23.5 | 207 | 122 | 3 | 3 | 5.46 | 0.94 | 3.44 | 1.76 | 4.76 | Y | N | Hypertension | Dalian |
| D07 | M | 63 | 168 | 71 | 25.2 | 145 | 95 | 1 | 1 | 4.97 | 0.82 | 4.31 | 2.65 | 6.01 | N | N | Hypertension | Dalian |
| D08 | M | 46 | 175 | 63 | 20.6 | 145 | 90 | 1 | 0 | 4.27 | 1.12 | 2.24 | 0.84 | 4.63 | N | N | Hypertension | Dalian |
| D09 | M | 45 | 154 | 60 | 25.3 | 166 | 104 | 2 | 3 | 12.5 | 0.96 | 2.98 | 2.73 | 4.53 | N | Y | Hypertension | Dalian |
| D10 | F | 62 | 162 | 64 | 24.4 | 173 | 105 | 2 | 1 | 14.35 | 0.9 | 3.74 | 2.92 | 5.6 | N | Y | Hypertension | Dalian |
| D11 | M | 73 | 168 | 61 | 21.6 | 176 | 103 | 2 | 0 | 4.22 | 1.31 | 2.2 | 1.87 | 3.85 | Y | N | Hypertension | Dalian |
| D12 | M | 73 | 169 | 76 | 26.6 | 161 | 97 | 2 | 0 | 5.05 | 1.05 | 3.22 | 2.05 | 5.22 | N | N | Hypertension | Dalian |
| D13 | M | 65 | 158 | 65 | 26 | 150 | 95 | 1 | 3 | 7.59 | 1.21 | 3.19 | 2.62 | 4.97 | N | Y | Hypertension | Dalian |
| D14 | F | 64 | 151 | 73 | 32 | 145 | 90 | 1 | 4 | 10.68 | 1.19 | 2.45 | 4.23 | 4.67 | N | Y | Hypertension | Dalian |
| D15 | M | 49 | 167 | 67 | 24 | 190 | 110 | 3 | 5 | 4.91 | 1.13 | 2.85 | 1.5 | 4.58 | N | N | Hypertension | Dalian |
| D16 | M | 61 | 163 | 55 | 20.7 | 140 | 90 | 1 | 0 | 5.09 | 1.07 | 2.91 | 1.31 | 4.47 | N | N | Hypertension | Dalian |
| D17 | M | 47 | 162 | 65 | 24.8 | 152 | 95 | 1 | 3 | 4.53 | 0.7 | 2.87 | 1.12 | 4.56 | N | N | Hypertension | Dalian |
| D18 | M | 55 | 164 | 52 | 19.3 | 140 | 90 | 1 | 2 | 8.71 | 1.59 | 2.55 | 3.71 | 6.24 | N | Y | Hypertension | Dalian |
| D19 | M | 49 | 162 | 54 | 20.6 | 182 | 107 | 3 | 6 | 4.74 | 1.54 | 2.95 | 3.31 | 4.95 | Y | N | Hypertension | Dalian |
| D20 | F | 59 | 155 | 61 | 25.4 | 160 | 100 | 2 | 7 | 8.62 | 1.18 | 3.02 | 3.35 | 4.8 | N | Y | Hypertension | Dalian |
| D21 | F | 55 | 170 | 73 | 25.3 | 180 | 95 | 3 | 5 | 4.33 | 1.28 | 2.55 | 0.87 | 5.74 | N | N | Hypertension | Dalian |
| D22 | F | 46 | 168 | 64 | 22.7 | 175 | 102 | 2 | 3 | 4.67 | 1.59 | 2.81 | 1.42 | 5.27 | N | N | Hypertension | Dalian |
| D23 | F | 51 | 155 | 50 | 20.8 | 180 | 110 | 3 | 6 | 4.65 | 1.36 | 1.72 | 0.71 | 3.36 | Y | N | Hypertension | Dalian |
| D24 | M | 45 | 153 | 59 | 25.2 | 160 | 90 | 2 | 5 | 8.32 | 1.16 | 2.62 | 0.76 | 4.16 | N | Y | Hypertension | Dalian |
| D25 | F | 62 | 166 | 70 | 25.4 | 150 | 90 | 1 | 2 | 5.06 | 0.92 | 3.26 | 0.45 | 5.88 | N | N | Hypertension | Dalian |
| D26 | F | 74 | 161 | 59 | 22.8 | 190 | 124 | 3 | 0 | 4.5 | 1.2 | 2.21 | 0.88 | 4.17 | Y | N | Hypertension | Dalian |
| D27 | F | 43 | 160 | 66 | 25.8 | 170 | 95 | 2 | 1 | 8.3 | 1.07 | 2.31 | 2.45 | 4.65 | N | Y | Hypertension | Dalian |
| D28 | M | 48 | 157 | 45 | 18.3 | 140 | 90 | 1 | 0 | 5.51 | 1.48 | 4.81 | 1.97 | 7.04 | Y | N | Hypertension | Dalian |
| D29 | M | 51 | 157 | 66 | 26.8 | 210 | 121 | 3 | 4 | 5.02 | 1.29 | 3.26 | 0.81 | 4.75 | N | N | Hypertension | Dalian |
| D30 | M | 58 | 161 | 72 | 27.8 | 141 | 90 | 1 | 0 | 5.66 | 1.24 | 3.07 | 1.32 | 5.46 | N | N | Hypertension | Dalian |
| D31 | M | 61 | 165 | 56 | 20.6 | 194 | 100 | 3 | 3 | 4.82 | 1.71 | 3.36 | 1.29 | 5.56 | N | N | Hypertension | Dalian |
| D32 | M | 54 | 173 | 76 | 25.4 | 145 | 90 | 1 | 4 | 4.79 | 0.89 | 2.37 | 0.97 | 4.21 | N | N | Hypertension | Dalian |
| D33 | M | 72 | 159 | 51 | 20.2 | 140 | 90 | 1 | 0 | 11.09 | 0.88 | 5.26 | 3.52 | 8.73 | N | Y | Hypertension | Dalian |
| D34 | M | 62 | 152 | 50 | 21.6 | 165 | 95 | 2 | 0 | 4.5 | 1.14 | 2.46 | 1.03 | 4.33 | N | N | Hypertension | Dalian |
| D35 | M | 54 | 163 | 68 | 25.6 | 195 | 121 | 3 | 1 | 5.6 | 1.49 | 2.95 | 1.37 | 5.54 | Y | N | Hypertension | Dalian |
| D36 | F | 52 | 169 | 58 | 20.3 | 185 | 105 | 3 | 2 | 5.65 | 1.45 | 3.32 | 1.13 | 4.98 | N | N | Hypertension | Dalian |
| D37 | F | 68 | 152 | 51 | 22.1 | 165 | 100 | 2 | 0 | 5.99 | 1.1 | 4.15 | 1.2 | 5.94 | N | N | Hypertension | Dalian |
| D38 | F | 62 | 180 | 70 | 21.6 | 145 | 100 | 1 | 6 | 6.78 | 0.81 | 4.11 | 2.55 | 5.7 | N | Y | Hypertension | Dalian |
| D39 | M | 44 | 169 | 72 | 25.2 | 149 | 98 | 1 | 0 | 4.9 | 0.62 | 1.79 | 2.23 | 3 | Y | N | Hypertension | Dalian |
| D40 | F | 68 | 175 | 80 | 26.1 | 195 | 115 | 3 | 0 | 7.93 | 1.03 | 3.95 | 1.76 | 5.53 | Y | Y | Hypertension | Dalian |
| D41 | M | 75 | 160 | 75 | 29.3 | 170 | 110 | 2 | 2 | 5.71 | 0.97 | 2.81 | 1.59 | 5.1 | N | N | Hypertension | Dalian |
| D42 | M | 50 | 172 | 70 | 23.7 | 180 | 100 | 3 | 8 | 5.14 | 1.49 | 3.64 | 1.26 | 3.98 | Y | N | Hypertension | Dalian |
| D43 | M | 46 | 164 | 55 | 20.4 | 165 | 101 | 2 | 0 | 6 | 0.65 | 1.38 | 1.23 | 2.68 | Y | N | Hypertension | Dalian |
| D44 | F | 54 | 170 | 63 | 21.8 | 161 | 107 | 2 | 4 | 4.73 | 1.31 | 3.43 | 1.79 | 5.47 | Y | N | Hypertension | Dalian |
| D45 | M | 58 | 170 | 55 | 19 | 140 | 85 | 1 | 1 | 5.73 | 1.3 | 3.16 | 0.98 | 5.44 | Y | N | Hypertension | Dalian |
| D46 | F | 70 | 158 | 59 | 23.6 | 165 | 92 | 2 | 0 | 3.13 | 1.03 | 2.45 | 1.2 | 5.27 | N | N | Hypertension | Dalian |
| D47 | F | 57 | 165 | 56 | 20.6 | 192 | 105 | 3 | 10 | 5 | 1.51 | 4.26 | 2.44 | 5.84 | N | N | Hypertension | Dalian |
| D48 | F | 47 | 153 | 56 | 23.9 | 200 | 110 | 3 | 2 | 4.66 | 1.02 | 3.31 | 1.44 | 4.94 | N | N | Hypertension | Dalian |
| D49 | F | 74 | 165 | 54 | 19.8 | 160 | 95 | 2 | 3 | 5.7 | 1.09 | 2.38 | 0.96 | 4.92 | N | N | Hypertension | Dalian |
| D50 | F | 45 | 155 | 60 | 25 | 140 | 90 | 1 | 0 | 9.52 | 1.25 | 3.13 | 2.51 | 5.25 | N | Y | Hypertension | Dalian |
| D51 | M | 49 | 170 | 65 | 22.5 | 180 | 124 | 3 | 4 | 5.61 | 1.37 | 3.53 | 1.36 | 5.54 | N | N | Hypertension | Dalian |
| D52 | M | 72 | 163 | 68 | 25.6 | 170 | 100 | 2 | 3 | 4.61 | 1.37 | 4.35 | 1.73 | 6.92 | Y | N | Hypertension | Dalian |
| D53 | F | 76 | 161 | 50 | 19.3 | 145 | 90 | 1 | 0 | 4.19 | 1.04 | 2.76 | 2.11 | 4.25 | N | N | Hypertension | Dalian |
| D54 | M | 64 | 156 | 66 | 27.1 | 145 | 95 | 1 | 0 | 12.93 | 0.96 | 3.15 | 2.8 | 5.29 | Y | Y | Hypertension | Dalian |
| D55 | F | 54 | 170 | 64 | 22.1 | 167 | 105 | 2 | 5 | 4.93 | 1.46 | 1.76 | 1.88 | 3.2 | Y | N | Hypertension | Dalian |
| D56 | M | 63 | 158 | 66 | 26.4 | 189 | 117 | 3 | 10 | 5.9 | 0.75 | 3.02 | 2.44 | 5.67 | N | Y | Hypertension | Dalian |
| D57 | F | 48 | 171 | 67 | 22.9 | 180 | 113 | 3 | 1 | 4.95 | 1.08 | 2.33 | 1.21 | 4.27 | N | N | Hypertension | Dalian |
| D58 | M | 49 | 160 | 68 | 26.6 | 160 | 98 | 2 | 2 | 7.93 | 0.96 | 3.06 | 3.01 | 4.23 | Y | Y | Hypertension | Dalian |
| D59 | M | 47 | 171 | 74 | 25.3 | 160 | 115 | 2 | 0 | 9.38 | 0.92 | 2.35 | 2.83 | 4.89 | Y | Y | Hypertension | Dalian |
| D60 | M | 57 | 171 | 64 | 21.9 | 140 | 90 | 1 | 0 | 5.43 | 1.15 | 3.6 | 1.47 | 5.01 | N | N | Hypertension | Dalian |
| H01 | F | 54 | 162 | 61 | 23.2 | 92 | 61 | - | - | 5.42 | 1.17 | 2.36 | 1.04 | 3.67 | N | N | Control | Dalian |
| H02 | F | 57 | 158 | 62 | 24.8 | 120 | 78 | - | - | 6.6 | 1.31 | 2.28 | 1.69 | 4.12 | N | Y | Control | Dalian |
| H03 | F | 48 | 168 | 58 | 20.5 | 110 | 70 | - | - | 5.41 | 0.89 | 2.89 | 1.99 | 5.06 | Y | N | Control | Dalian |
| H04 | M | 54 | 155 | 65 | 27.1 | 117 | 73 | - | - | 5.05 | 1.2 | 3.07 | 0.68 | 4.4 | N | N | Control | Dalian |
| H05 | M | 64 | 154 | 50 | 21.1 | 116 | 60 | - | - | 10.12 | 1.05 | 2.97 | 2.03 | 7.5 | N | Y | Control | Dalian |
| H06 | F | 63 | 155 | 44 | 18.3 | 110 | 60 | - | - | 8.94 | 1.24 | 2.32 | 1.43 | 3.24 | N | Y | Control | Dalian |
| H07 | F | 46 | 147 | 52 | 24.1 | 112 | 80 | - | - | 5.61 | 1.31 | 3.04 | 1.12 | 5.12 | N | N | Control | Dalian |
| H08 | F | 57 | 165 | 60 | 22 | 110 | 60 | - | - | 9.2 | 1.88 | 2.87 | 1.3 | 5.83 | N | Y | Control | Dalian |
| H09 | M | 67 | 160 | 68 | 26.6 | 100 | 70 | - | - | 4.32 | 0.9 | 2.37 | 2.58 | 4.99 | Y | N | Control | Dalian |
| H10 | M | 44 | 148 | 43 | 25 | 110 | 70 | - | - | 5.7 | 1 | 2.45 | 1.31 | 3.7 | Y | N | Control | Dalian |
| H11 | M | 58 | 170 | 61 | 21.1 | 102 | 61 | - | - | 3.9 | 1.35 | 2.54 | 0.6 | 3.94 | Y | N | Control | Dalian |
| H12 | F | 71 | 155 | 64 | 26.6 | 118 | 65 | - | - | 4.76 | 0.76 | 2.08 | 1.79 | 3.79 | Y | N | Control | Dalian |
| H13 | F | 50 | 158 | 48 | 19.2 | 120 | 79 | - | - | 4.34 | 1.45 | 2.36 | 0.42 | 4.32 | N | N | Control | Dalian |
| H14 | M | 70 | 170 | 67 | 23.2 | 115 | 64 | - | - | 4.92 | 1.48 | 3.12 | 0.65 | 5.58 | N | N | Control | Dalian |
| H15 | M | 72 | 173 | 65 | 21.7 | 98 | 68 | - | - | 5.29 | 1.1 | 3.03 | 1.02 | 4.67 | Y | N | Control | Dalian |
| H16 | F | 72 | 151 | 54 | 23.7 | 110 | 73 | - | - | 12.38 | 1.29 | 4.11 | 1.88 | 5.93 | N | Y | Control | Dalian |
| H17 | F | 51 | 170 | 71 | 24.6 | 110 | 67 | - | - | 5.23 | 1.13 | 2.9 | 0.68 | 4.76 | N | N | Control | Dalian |
| H18 | M | 46 | 157 | 60 | 24.3 | 110 | 80 | - | - | 4.23 | 1.15 | 3.83 | 0.84 | 4.82 | Y | N | Control | Dalian |
| H19 | M | 50 | 160 | 63 | 24.6 | 120 | 75 | - | - | 5.32 | 1.05 | 3.04 | 1.5 | 4.32 | Y | N | Control | Dalian |
| H20 | F | 69 | 160 | 55 | 21.5 | 115 | 65 | - | - | 5.05 | 1.16 | 4.14 | 1.49 | 6.16 | N | N | Control | Dalian |
| H21 | M | 68 | 168 | 59 | 20.9 | 115 | 75 | - | - | 8.39 | 0.99 | 2.66 | 1.48 | 6.48 | Y | Y | Control | Dalian |
| H22 | M | 62 | 165 | 50 | 18.4 | 110 | 70 | - | - | 6.99 | 1.11 | 2.56 | 2.31 | 4.9 | N | Y | Control | Dalian |
| H23 | M | 42 | 167 | 75 | 26.9 | 114 | 82 | - | - | 7.07 | 1.3 | 3.83 | 2.04 | 4.72 | Y | Y | Control | Dalian |
| H24 | M | 60 | 169 | 64 | 22.4 | 116 | 80 | - | - | 5.66 | 1.28 | 3.22 | 0.93 | 5.25 | N | N | Control | Dalian |
| H25 | F | 43 | 164 | 58 | 21.6 | 119 | 79 | - | - | 4.92 | 0.96 | 3.42 | 2.08 | 5.65 | Y | N | Control | Dalian |
| H26 | F | 55 | 148 | 52 | 23.7 | 105 | 65 | - | - | 4.38 | 1.12 | 5.15 | 2.24 | 7.13 | N | N | Control | Dalian |
| H27 | F | 57 | 162 | 56 | 21.3 | 112 | 75 | - | - | 4.94 | 1.09 | 3.82 | 1.53 | 5.93 | N | N | Control | Dalian |
| H28 | M | 50 | 172 | 80 | 27 | 120 | 76 | - | - | 4.96 | 1.94 | 4.39 | 0.8 | 7.07 | N | N | Control | Dalian |
| H29 | M | 53 | 160 | 45 | 17.6 | 112 | 80 | - | - | 8.9 | 0.98 | 2.88 | 2.3 | 3.96 | Y | Y | Control | Dalian |
| H30 | M | 65 | 164 | 62 | 23.1 | 110 | 80 | - | - | 6.68 | 1.38 | 2.17 | 0.63 | 3.7 | N | Y | Control | Dalian |
| H31 | M | 54 | 166 | 68 | 24.7 | 118 | 72 | - | - | 8.3 | 1.17 | 2.64 | 1.37 | 4.13 | N | Y | Control | Dalian |
| H32 | F | 71 | 168 | 78 | 27.6 | 110 | 83 | - | - | 4.85 | 1.31 | 3.13 | 1.26 | 5.51 | N | N | Control | Dalian |
| H33 | F | 46 | 172 | 75 | 25.4 | 111 | 66 | - | - | 4.26 | 1.28 | 2.12 | 1.11 | 6.13 | N | N | Control | Dalian |
| H34 | F | 64 | 172 | 73 | 24.7 | 108 | 64 | - | - | 8.5 | 1.2 | 2.03 | 0.69 | 3.94 | N | Y | Control | Dalian |
| H35 | M | 45 | 150 | 50 | 22.2 | 112 | 72 | - | - | 9.11 | 1.46 | 2.59 | 2.5 | 4.81 | Y | Y | Control | Dalian |
| H36 | M | 56 | 155 | 50 | 20.8 | 110 | 70 | - | - | 7.83 | 0.99 | 4.03 | 2.35 | 5.31 | Y | Y | Control | Dalian |
| H37 | M | 56 | 156 | 60 | 24.9 | 107 | 63 | - | - | 5.62 | 0.96 | 2.43 | 2 | 5.04 | N | N | Control | Dalian |
| H38 | F | 43 | 153 | 54 | 23.1 | 118 | 74 | - | - | 5.05 | 1.61 | 4.14 | 1.96 | 5.99 | N | N | Control | Dalian |
| H39 | M | 53 | 178 | 78 | 24.6 | 109 | 66 | - | - | 4.27 | 1.44 | 2.52 | 0.97 | 4.58 | Y | N | Control | Dalian |
| H40 | M | 62 | 160 | 59 | 23 | 94 | 61 | - | - | 7.2 | 1.33 | 2.41 | 0.56 | 4.03 | N | Y | Control | Dalian |
| H41 | F | 56 | 158 | 74 | 29.6 | 109 | 75 | - | - | 6.96 | 1.74 | 3.52 | 2.26 | 4.49 | N | Y | Control | Dalian |
| H42 | F | 58 | 155 | 46 | 19.1 | 120 | 70 | - | - | 8.28 | 1.03 | 3.83 | 2.62 | 5.65 | Y | Y | Control | Dalian |
| H43 | M | 58 | 163 | 54 | 20.3 | 116 | 78 | - | - | 4.23 | 2.02 | 2.25 | 0.45 | 4.95 | Y | N | Control | Dalian |
| H44 | M | 54 | 150 | 50 | 22.2 | 110 | 76 | - | - | 5.16 | 1.93 | 4.14 | 1.13 | 6.21 | Y | N | Control | Dalian |
| H45 | F | 60 | 164 | 68 | 25.3 | 112 | 80 | - | - | 4.48 | 1.43 | 2.29 | 0.96 | 4.13 | N | N | Control | Dalian |
| H46 | F | 50 | 150 | 48 | 21.3 | 112 | 68 | - | - | 4.23 | 0.97 | 2.75 | 1.61 | 4.06 | N | N | Control | Dalian |
| H47 | M | 46 | 170 | 62 | 21.5 | 110 | 70 | - | - | 14.06 | 1 | 3.05 | 2.08 | 5.44 | Y | Y | Control | Dalian |
| H48 | F | 43 | 175 | 72 | 23.5 | 107 | 80 | - | - | 5.02 | 0.72 | 2.83 | 1.26 | 3.59 | N | N | Control | Dalian |
| H49 | F | 59 | 165 | 71 | 26.1 | 110 | 70 | - | - | 9.72 | 1.01 | 3.95 | 2.18 | 6.57 | N | Y | Control | Dalian |
| H50 | M | 48 | 160 | 63 | 24.6 | 116 | 69 | - | - | 4.78 | 1.25 | 2.5 | 1.75 | 4.29 | Y | N | Control | Dalian |
| H51 | F | 55 | 150 | 58 | 25.8 | 105 | 80 | - | - | 5.51 | 0.92 | 3.91 | 2.12 | 5.24 | Y | N | Control | Dalian |
| H52 | M | 71 | 171 | 74 | 25.3 | 110 | 80 | - | - | 5.69 | 1.45 | 2.69 | 0.59 | 5.75 | N | N | Control | Dalian |
| H53 | M | 49 | 180 | 90 | 27.8 | 110 | 70 | - | - | 7.73 | 0.77 | 3.9 | 3.66 | 6.01 | Y | Y | Control | Dalian |
| H54 | F | 51 | 168 | 75 | 26.6 | 115 | 70 | - | - | 5.12 | 1.31 | 3.06 | 1.72 | 4.63 | N | N | Control | Dalian |
| H55 | M | 55 | 167 | 55 | 19.7 | 106 | 72 | - | - | 5.29 | 1.02 | 2.06 | 2.43 | 6.01 | N | N | Control | Dalian |
| H56 | F | 46 | 150 | 50 | 22.2 | 112 | 62 | - | - | 5.53 | 1.47 | 2.79 | 1.47 | 6.4 | N | N | Control | Dalian |
| H57 | F | 48 | 152 | 51 | 22.1 | 118 | 66 | - | - | 5.5 | 1.07 | 3.7 | 1.75 | 5.67 | N | N | Control | Dalian |
| H58 | M | 60 | 168 | 71 | 25.2 | 110 | 60 | - | - | 4.74 | 0.99 | 3.29 | 2.35 | 5.21 | N | N | Control | Dalian |
| H59 | M | 52 | 168 | 65 | 23 | 95 | 65 | - | - | 5.18 | 1.26 | 3.61 | 0.81 | 5.17 | Y | N | Control | Dalian |
| H60 | M | 70 | 158 | 68 | 27.2 | 110 | 80 | - | - | 4.4 | 1.57 | 2.15 | 0.78 | 4.43 | Y | N | Control | Dalian |
| ERR1398129 | NA | NA | NA | NA | NA | NA | NA | NA | NA | NA | NA | NA | NA | NA | NA | NA | Control | Beijing |
| ERR1398206 | NA | NA | NA | NA | NA | NA | NA | NA | NA | NA | NA | NA | NA | NA | NA | NA | Control | Beijing |
| ERR1398173 | NA | NA | NA | NA | NA | NA | NA | NA | NA | NA | NA | NA | NA | NA | NA | NA | Control | Beijing |
| ERR1398075 | NA | NA | NA | NA | NA | NA | NA | NA | NA | NA | NA | NA | NA | NA | NA | NA | Control | Beijing |
| ERR1398114 | NA | NA | NA | NA | NA | NA | NA | NA | NA | NA | NA | NA | NA | NA | NA | NA | Control | Beijing |
| ERR1398242 | NA | NA | NA | NA | NA | NA | NA | NA | NA | NA | NA | NA | NA | NA | NA | NA | Control | Beijing |
| ERR1398164 | NA | NA | NA | NA | NA | NA | NA | NA | NA | NA | NA | NA | NA | NA | NA | NA | Control | Beijing |
| ERR1398224 | NA | NA | NA | NA | NA | NA | NA | NA | NA | NA | NA | NA | NA | NA | NA | NA | Control | Beijing |
| ERR1398243 | NA | NA | NA | NA | NA | NA | NA | NA | NA | NA | NA | NA | NA | NA | NA | NA | Control | Beijing |
| ERR1398248 | NA | NA | NA | NA | NA | NA | NA | NA | NA | NA | NA | NA | NA | NA | NA | NA | Control | Beijing |
| ERR1398100 | NA | NA | NA | NA | NA | NA | NA | NA | NA | NA | NA | NA | NA | NA | NA | NA | Control | Beijing |
| ERR1398237 | NA | NA | NA | NA | NA | NA | NA | NA | NA | NA | NA | NA | NA | NA | NA | NA | Control | Beijing |
| ERR1398101 | NA | NA | NA | NA | NA | NA | NA | NA | NA | NA | NA | NA | NA | NA | NA | NA | Control | Beijing |
| ERR1398214 | NA | NA | NA | NA | NA | NA | NA | NA | NA | NA | NA | NA | NA | NA | NA | NA | Control | Beijing |
| ERR1398263 | NA | NA | NA | NA | NA | NA | NA | NA | NA | NA | NA | NA | NA | NA | NA | NA | Control | Beijing |
| ERR1398161 | NA | NA | NA | NA | NA | NA | NA | NA | NA | NA | NA | NA | NA | NA | NA | NA | Control | Beijing |
| ERR1398178 | NA | NA | NA | NA | NA | NA | NA | NA | NA | NA | NA | NA | NA | NA | NA | NA | Control | Beijing |
| ERR1398148 | NA | NA | NA | NA | NA | NA | NA | NA | NA | NA | NA | NA | NA | NA | NA | NA | Control | Beijing |
| ERR1398078 | NA | NA | NA | NA | NA | NA | NA | NA | NA | NA | NA | NA | NA | NA | NA | NA | Control | Beijing |
| ERR1398127 | NA | NA | NA | NA | NA | NA | NA | NA | NA | NA | NA | NA | NA | NA | NA | NA | Control | Beijing |
| ERR1398180 | NA | NA | NA | NA | NA | NA | NA | NA | NA | NA | NA | NA | NA | NA | NA | NA | Control | Beijing |
| ERR1398144 | NA | NA | NA | NA | NA | NA | NA | NA | NA | NA | NA | NA | NA | NA | NA | NA | Control | Beijing |
| ERR1398089 | NA | NA | NA | NA | NA | NA | NA | NA | NA | NA | NA | NA | NA | NA | NA | NA | Control | Beijing |
| ERR1398205 | NA | NA | NA | NA | NA | NA | NA | NA | NA | NA | NA | NA | NA | NA | NA | NA | Control | Beijing |
| ERR1398109 | NA | NA | NA | NA | NA | NA | NA | NA | NA | NA | NA | NA | NA | NA | NA | NA | Control | Beijing |
| ERR1398169 | NA | NA | NA | NA | NA | NA | NA | NA | NA | NA | NA | NA | NA | NA | NA | NA | Control | Beijing |
| ERR1398126 | NA | NA | NA | NA | NA | NA | NA | NA | NA | NA | NA | NA | NA | NA | NA | NA | Control | Beijing |
| ERR1398083 | NA | NA | NA | NA | NA | NA | NA | NA | NA | NA | NA | NA | NA | NA | NA | NA | Control | Beijing |
| ERR1398138 | NA | NA | NA | NA | NA | NA | NA | NA | NA | NA | NA | NA | NA | NA | NA | NA | Control | Beijing |
| ERR1398244 | NA | NA | NA | NA | NA | NA | NA | NA | NA | NA | NA | NA | NA | NA | NA | NA | Control | Beijing |
| ERR1398223 | NA | NA | NA | NA | NA | NA | NA | NA | NA | NA | NA | NA | NA | NA | NA | NA | Control | Beijing |
| ERR1398152 | NA | NA | NA | NA | NA | NA | NA | NA | NA | NA | NA | NA | NA | NA | NA | NA | Control | Beijing |
| ERR1398192 | NA | NA | NA | NA | NA | NA | NA | NA | NA | NA | NA | NA | NA | NA | NA | NA | Control | Beijing |
| ERR1398217 | NA | NA | NA | NA | NA | NA | NA | NA | NA | NA | NA | NA | NA | NA | NA | NA | Control | Beijing |
| ERR1398113 | NA | NA | NA | NA | NA | NA | NA | NA | NA | NA | NA | NA | NA | NA | NA | NA | Control | Beijing |
| ERR1398118 | NA | NA | NA | NA | NA | NA | NA | NA | NA | NA | NA | NA | NA | NA | NA | NA | Control | Beijing |
| ERR1398213 | NA | NA | NA | NA | NA | NA | NA | NA | NA | NA | NA | NA | NA | NA | NA | NA | Control | Beijing |
| ERR1398253 | NA | NA | NA | NA | NA | NA | NA | NA | NA | NA | NA | NA | NA | NA | NA | NA | Control | Beijing |
| ERR1398218 | NA | NA | NA | NA | NA | NA | NA | NA | NA | NA | NA | NA | NA | NA | NA | NA | Control | Beijing |
| ERR1398198 | NA | NA | NA | NA | NA | NA | NA | NA | NA | NA | NA | NA | NA | NA | NA | NA | Control | Beijing |
| ERR1398153 | NA | NA | NA | NA | NA | NA | NA | NA | NA | NA | NA | NA | NA | NA | NA | NA | Control | Beijing |
| ERR1398151 | NA | NA | NA | NA | NA | NA | NA | NA | NA | NA | NA | NA | NA | NA | NA | NA | Hypertension | Beijing |
| ERR1398079 | NA | NA | NA | NA | NA | NA | NA | NA | NA | NA | NA | NA | NA | NA | NA | NA | Hypertension | Beijing |
| ERR1398103 | NA | NA | NA | NA | NA | NA | NA | NA | NA | NA | NA | NA | NA | NA | NA | NA | Hypertension | Beijing |
| ERR1398099 | NA | NA | NA | NA | NA | NA | NA | NA | NA | NA | NA | NA | NA | NA | NA | NA | Hypertension | Beijing |
| ERR1398197 | NA | NA | NA | NA | NA | NA | NA | NA | NA | NA | NA | NA | NA | NA | NA | NA | Hypertension | Beijing |
| ERR1398245 | NA | NA | NA | NA | NA | NA | NA | NA | NA | NA | NA | NA | NA | NA | NA | NA | Hypertension | Beijing |
| ERR1398179 | NA | NA | NA | NA | NA | NA | NA | NA | NA | NA | NA | NA | NA | NA | NA | NA | Hypertension | Beijing |
| ERR1398228 | NA | NA | NA | NA | NA | NA | NA | NA | NA | NA | NA | NA | NA | NA | NA | NA | Hypertension | Beijing |
| ERR1398170 | NA | NA | NA | NA | NA | NA | NA | NA | NA | NA | NA | NA | NA | NA | NA | NA | Hypertension | Beijing |
| ERR1398239 | NA | NA | NA | NA | NA | NA | NA | NA | NA | NA | NA | NA | NA | NA | NA | NA | Hypertension | Beijing |
| ERR1398229 | NA | NA | NA | NA | NA | NA | NA | NA | NA | NA | NA | NA | NA | NA | NA | NA | Hypertension | Beijing |
| ERR1398232 | NA | NA | NA | NA | NA | NA | NA | NA | NA | NA | NA | NA | NA | NA | NA | NA | Hypertension | Beijing |
| ERR1398208 | NA | NA | NA | NA | NA | NA | NA | NA | NA | NA | NA | NA | NA | NA | NA | NA | Hypertension | Beijing |
| ERR1398069 | NA | NA | NA | NA | NA | NA | NA | NA | NA | NA | NA | NA | NA | NA | NA | NA | Hypertension | Beijing |
| ERR1398182 | NA | NA | NA | NA | NA | NA | NA | NA | NA | NA | NA | NA | NA | NA | NA | NA | Hypertension | Beijing |
| ERR1398117 | NA | NA | NA | NA | NA | NA | NA | NA | NA | NA | NA | NA | NA | NA | NA | NA | Hypertension | Beijing |
| ERR1398221 | NA | NA | NA | NA | NA | NA | NA | NA | NA | NA | NA | NA | NA | NA | NA | NA | Hypertension | Beijing |
| ERR1398174 | NA | NA | NA | NA | NA | NA | NA | NA | NA | NA | NA | NA | NA | NA | NA | NA | Hypertension | Beijing |
| ERR1398225 | NA | NA | NA | NA | NA | NA | NA | NA | NA | NA | NA | NA | NA | NA | NA | NA | Hypertension | Beijing |
| ERR1398149 | NA | NA | NA | NA | NA | NA | NA | NA | NA | NA | NA | NA | NA | NA | NA | NA | Hypertension | Beijing |
| ERR1398200 | NA | NA | NA | NA | NA | NA | NA | NA | NA | NA | NA | NA | NA | NA | NA | NA | Hypertension | Beijing |
| ERR1398216 | NA | NA | NA | NA | NA | NA | NA | NA | NA | NA | NA | NA | NA | NA | NA | NA | Hypertension | Beijing |
| ERR1398131 | NA | NA | NA | NA | NA | NA | NA | NA | NA | NA | NA | NA | NA | NA | NA | NA | Hypertension | Beijing |
| ERR1398119 | NA | NA | NA | NA | NA | NA | NA | NA | NA | NA | NA | NA | NA | NA | NA | NA | Hypertension | Beijing |
| ERR1398110 | NA | NA | NA | NA | NA | NA | NA | NA | NA | NA | NA | NA | NA | NA | NA | NA | Hypertension | Beijing |
| ERR1398166 | NA | NA | NA | NA | NA | NA | NA | NA | NA | NA | NA | NA | NA | NA | NA | NA | Hypertension | Beijing |
| ERR1398227 | NA | NA | NA | NA | NA | NA | NA | NA | NA | NA | NA | NA | NA | NA | NA | NA | Hypertension | Beijing |
| ERR1398207 | NA | NA | NA | NA | NA | NA | NA | NA | NA | NA | NA | NA | NA | NA | NA | NA | Hypertension | Beijing |
| ERR1398082 | NA | NA | NA | NA | NA | NA | NA | NA | NA | NA | NA | NA | NA | NA | NA | NA | Hypertension | Beijing |
| ERR1398146 | NA | NA | NA | NA | NA | NA | NA | NA | NA | NA | NA | NA | NA | NA | NA | NA | Hypertension | Beijing |
| ERR1398128 | NA | NA | NA | NA | NA | NA | NA | NA | NA | NA | NA | NA | NA | NA | NA | NA | Hypertension | Beijing |
| ERR1398204 | NA | NA | NA | NA | NA | NA | NA | NA | NA | NA | NA | NA | NA | NA | NA | NA | Hypertension | Beijing |
| ERR1398199 | NA | NA | NA | NA | NA | NA | NA | NA | NA | NA | NA | NA | NA | NA | NA | NA | Hypertension | Beijing |
| ERR1398088 | NA | NA | NA | NA | NA | NA | NA | NA | NA | NA | NA | NA | NA | NA | NA | NA | Hypertension | Beijing |
| ERR1398093 | NA | NA | NA | NA | NA | NA | NA | NA | NA | NA | NA | NA | NA | NA | NA | NA | Hypertension | Beijing |
| ERR1398137 | NA | NA | NA | NA | NA | NA | NA | NA | NA | NA | NA | NA | NA | NA | NA | NA | Hypertension | Beijing |
| ERR1398143 | NA | NA | NA | NA | NA | NA | NA | NA | NA | NA | NA | NA | NA | NA | NA | NA | Hypertension | Beijing |
| ERR1398203 | NA | NA | NA | NA | NA | NA | NA | NA | NA | NA | NA | NA | NA | NA | NA | NA | Hypertension | Beijing |
| ERR1398260 | NA | NA | NA | NA | NA | NA | NA | NA | NA | NA | NA | NA | NA | NA | NA | NA | Hypertension | Beijing |
| ERR1398163 | NA | NA | NA | NA | NA | NA | NA | NA | NA | NA | NA | NA | NA | NA | NA | NA | Hypertension | Beijing |
| ERR1398247 | NA | NA | NA | NA | NA | NA | NA | NA | NA | NA | NA | NA | NA | NA | NA | NA | Hypertension | Beijing |
| ERR1398238 | NA | NA | NA | NA | NA | NA | NA | NA | NA | NA | NA | NA | NA | NA | NA | NA | Hypertension | Beijing |
| ERR1398236 | NA | NA | NA | NA | NA | NA | NA | NA | NA | NA | NA | NA | NA | NA | NA | NA | Hypertension | Beijing |
| ERR1398254 | NA | NA | NA | NA | NA | NA | NA | NA | NA | NA | NA | NA | NA | NA | NA | NA | Hypertension | Beijing |
| ERR1398120 | NA | NA | NA | NA | NA | NA | NA | NA | NA | NA | NA | NA | NA | NA | NA | NA | Hypertension | Beijing |
| ERR1398211 | NA | NA | NA | NA | NA | NA | NA | NA | NA | NA | NA | NA | NA | NA | NA | NA | Hypertension | Beijing |
| ERR1398097 | NA | NA | NA | NA | NA | NA | NA | NA | NA | NA | NA | NA | NA | NA | NA | NA | Hypertension | Beijing |
| ERR1398184 | NA | NA | NA | NA | NA | NA | NA | NA | NA | NA | NA | NA | NA | NA | NA | NA | Hypertension | Beijing |
| ERR1398090 | NA | NA | NA | NA | NA | NA | NA | NA | NA | NA | NA | NA | NA | NA | NA | NA | Hypertension | Beijing |
| ERR1398132 | NA | NA | NA | NA | NA | NA | NA | NA | NA | NA | NA | NA | NA | NA | NA | NA | Hypertension | Beijing |
| ERR1398220 | NA | NA | NA | NA | NA | NA | NA | NA | NA | NA | NA | NA | NA | NA | NA | NA | Hypertension | Beijing |
| ERR1398115 | NA | NA | NA | NA | NA | NA | NA | NA | NA | NA | NA | NA | NA | NA | NA | NA | Hypertension | Beijing |
| ERR1398262 | NA | NA | NA | NA | NA | NA | NA | NA | NA | NA | NA | NA | NA | NA | NA | NA | Hypertension | Beijing |
| ERR1398123 | NA | NA | NA | NA | NA | NA | NA | NA | NA | NA | NA | NA | NA | NA | NA | NA | Hypertension | Beijing |
| ERR1398095 | NA | NA | NA | NA | NA | NA | NA | NA | NA | NA | NA | NA | NA | NA | NA | NA | Hypertension | Beijing |
| ERR1398181 | NA | NA | NA | NA | NA | NA | NA | NA | NA | NA | NA | NA | NA | NA | NA | NA | Hypertension | Beijing |
| ERR1398177 | NA | NA | NA | NA | NA | NA | NA | NA | NA | NA | NA | NA | NA | NA | NA | NA | Hypertension | Beijing |
| ERR1398104 | NA | NA | NA | NA | NA | NA | NA | NA | NA | NA | NA | NA | NA | NA | NA | NA | Hypertension | Beijing |
| ERR1398125 | NA | NA | NA | NA | NA | NA | NA | NA | NA | NA | NA | NA | NA | NA | NA | NA | Hypertension | Beijing |
| ERR1398076 | NA | NA | NA | NA | NA | NA | NA | NA | NA | NA | NA | NA | NA | NA | NA | NA | Hypertension | Beijing |
| ERR1398233 | NA | NA | NA | NA | NA | NA | NA | NA | NA | NA | NA | NA | NA | NA | NA | NA | Hypertension | Beijing |
| ERR1398130 | NA | NA | NA | NA | NA | NA | NA | NA | NA | NA | NA | NA | NA | NA | NA | NA | Hypertension | Beijing |
| ERR1398261 | NA | NA | NA | NA | NA | NA | NA | NA | NA | NA | NA | NA | NA | NA | NA | NA | Hypertension | Beijing |
| ERR1398172 | NA | NA | NA | NA | NA | NA | NA | NA | NA | NA | NA | NA | NA | NA | NA | NA | Hypertension | Beijing |
| ERR1398191 | NA | NA | NA | NA | NA | NA | NA | NA | NA | NA | NA | NA | NA | NA | NA | NA | Hypertension | Beijing |
| ERR1398134 | NA | NA | NA | NA | NA | NA | NA | NA | NA | NA | NA | NA | NA | NA | NA | NA | Hypertension | Beijing |
| ERR1398068 | NA | NA | NA | NA | NA | NA | NA | NA | NA | NA | NA | NA | NA | NA | NA | NA | Hypertension | Beijing |
| ERR1398124 | NA | NA | NA | NA | NA | NA | NA | NA | NA | NA | NA | NA | NA | NA | NA | NA | Hypertension | Beijing |
| ERR1398107 | NA | NA | NA | NA | NA | NA | NA | NA | NA | NA | NA | NA | NA | NA | NA | NA | Hypertension | Beijing |
| ERR1398250 | NA | NA | NA | NA | NA | NA | NA | NA | NA | NA | NA | NA | NA | NA | NA | NA | Hypertension | Beijing |
| ERR1398258 | NA | NA | NA | NA | NA | NA | NA | NA | NA | NA | NA | NA | NA | NA | NA | NA | Hypertension | Beijing |
| ERR1398159 | NA | NA | NA | NA | NA | NA | NA | NA | NA | NA | NA | NA | NA | NA | NA | NA | Hypertension | Beijing |
| ERR1398105 | NA | NA | NA | NA | NA | NA | NA | NA | NA | NA | NA | NA | NA | NA | NA | NA | Hypertension | Beijing |
| ERR1398202 | NA | NA | NA | NA | NA | NA | NA | NA | NA | NA | NA | NA | NA | NA | NA | NA | Hypertension | Beijing |
| ERR1398077 | NA | NA | NA | NA | NA | NA | NA | NA | NA | NA | NA | NA | NA | NA | NA | NA | Hypertension | Beijing |
| ERR1398235 | NA | NA | NA | NA | NA | NA | NA | NA | NA | NA | NA | NA | NA | NA | NA | NA | Hypertension | Beijing |
| ERR1398116 | NA | NA | NA | NA | NA | NA | NA | NA | NA | NA | NA | NA | NA | NA | NA | NA | Hypertension | Beijing |
| ERR1398256 | NA | NA | NA | NA | NA | NA | NA | NA | NA | NA | NA | NA | NA | NA | NA | NA | Hypertension | Beijing |
| ERR1398189 | NA | NA | NA | NA | NA | NA | NA | NA | NA | NA | NA | NA | NA | NA | NA | NA | Hypertension | Beijing |
| ERR1398073 | NA | NA | NA | NA | NA | NA | NA | NA | NA | NA | NA | NA | NA | NA | NA | NA | Hypertension | Beijing |
| ERR1398145 | NA | NA | NA | NA | NA | NA | NA | NA | NA | NA | NA | NA | NA | NA | NA | NA | Hypertension | Beijing |
| ERR1398230 | NA | NA | NA | NA | NA | NA | NA | NA | NA | NA | NA | NA | NA | NA | NA | NA | Hypertension | Beijing |
| ERR1398185 | NA | NA | NA | NA | NA | NA | NA | NA | NA | NA | NA | NA | NA | NA | NA | NA | Hypertension | Beijing |
| ERR1398168 | NA | NA | NA | NA | NA | NA | NA | NA | NA | NA | NA | NA | NA | NA | NA | NA | Hypertension | Beijing |
| ERR1398142 | NA | NA | NA | NA | NA | NA | NA | NA | NA | NA | NA | NA | NA | NA | NA | NA | Hypertension | Beijing |
| ERR1398135 | NA | NA | NA | NA | NA | NA | NA | NA | NA | NA | NA | NA | NA | NA | NA | NA | Hypertension | Beijing |
| ERR1398251 | NA | NA | NA | NA | NA | NA | NA | NA | NA | NA | NA | NA | NA | NA | NA | NA | Hypertension | Beijing |
| ERR1398186 | NA | NA | NA | NA | NA | NA | NA | NA | NA | NA | NA | NA | NA | NA | NA | NA | Hypertension | Beijing |
| ERR1398112 | NA | NA | NA | NA | NA | NA | NA | NA | NA | NA | NA | NA | NA | NA | NA | NA | Hypertension | Beijing |
| ERR1398122 | NA | NA | NA | NA | NA | NA | NA | NA | NA | NA | NA | NA | NA | NA | NA | NA | Hypertension | Beijing |
| ERR1398176 | NA | NA | NA | NA | NA | NA | NA | NA | NA | NA | NA | NA | NA | NA | NA | NA | Hypertension | Beijing |
| ERR1398252 | NA | NA | NA | NA | NA | NA | NA | NA | NA | NA | NA | NA | NA | NA | NA | NA | Hypertension | Beijing |
| ERR1398136 | NA | NA | NA | NA | NA | NA | NA | NA | NA | NA | NA | NA | NA | NA | NA | NA | Hypertension | Beijing |
| ERR1398085 | NA | NA | NA | NA | NA | NA | NA | NA | NA | NA | NA | NA | NA | NA | NA | NA | Hypertension | Beijing |
| ERR1398154 | NA | NA | NA | NA | NA | NA | NA | NA | NA | NA | NA | NA | NA | NA | NA | NA | Hypertension | Beijing |
| ERR1398212 | NA | NA | NA | NA | NA | NA | NA | NA | NA | NA | NA | NA | NA | NA | NA | NA | Hypertension | Beijing |
| ERR1398157 | NA | NA | NA | NA | NA | NA | NA | NA | NA | NA | NA | NA | NA | NA | NA | NA | Hypertension | Beijing |
| ERR1398162 | NA | NA | NA | NA | NA | NA | NA | NA | NA | NA | NA | NA | NA | NA | NA | NA | Hypertension | Beijing |
| ERR1398226 | NA | NA | NA | NA | NA | NA | NA | NA | NA | NA | NA | NA | NA | NA | NA | NA | Hypertension | Beijing |
| *Stage I: current blood pressure 140/90 ~ 159/99; stage II: current blood pressure 160/100 ~ 179~109; stage III: current blood pressure >180/110. | | | | | | | | | | | | | | | | | | |
