# Supplementary material for: Cross-cohort microbiome-wide study reveals consistent alterations in the gut bacteriome, but not the gut mycobiome, in patients with hypertension
Source: mSystems. 2025 Aug 15;10(9):e00657-25. doi: 10.1128/msystems.00657-25 (PMC12455987; doi:10.1128/msystems.00657-25)
Supplement: Table S2 — Detailed information of 61 gut bacterial signatures. [file msystems.00657-25-s0002.doc]

| **Table S2| Detailed information of 61 gut bacterial signatures between HTN patients and healthy controls.** |  |  |  |  |  |  |
| --- | --- | --- | --- | --- | --- | --- |
|  |  |  |  |  |  |  |
| **Bacterial signature** | **Enriched in (cohort 1)** | **P-value (cohort 1)** | **Enriched in (cohort 2)** | **P-value (cohort 2)** | **P-value (two cohort merged)** | **Combined P-value** |
| *Amedibacterium intestinale* | Control | 0.029824949 | Control | 0.207145566 | 0.02818781 | 0.037604546 |
| *Bacteroides intestinalis* | Disease | 0.375181586 | Disease | 0.005152596 | 0.003641738 | 0.014012696 |
| *Bacteroides nordii* | Disease | 0.838690133 | Disease | 0.002661892 | 0.012947736 | 0.015861108 |
| *Butyricimonas virosa* | Disease | 0.004468364 | Disease | 0.944586811 | 0.015553476 | 0.027298768 |
| *Candidatus Avimonas narfia* | Disease | 0.169746694 | Disease | 0.025886026 | 0.006998837 | 0.028242868 |
| *Candidatus Cibiobacter qucibialis* | Disease | 0.001281755 | Disease | 0.333617652 | 0.002316313 | 0.003744757 |
| *Candidatus Metaruminococcus caecorum* | Disease | 0.133144138 | Disease | 0.035490962 | 0.009124276 | 0.030029059 |
| *Clostridia bacterium* | Disease | 0.379667201 | Disease | 0.018086756 | 0.004692918 | 0.041071461 |
| *Clostridia unclassified SGB6276* | Disease | 0.011317952 | Disease | 0.327980893 | 0.000594173 | 0.024485439 |
| *Clostridiaceae bacterium* | Disease | 0.055493647 | Disease | 0.01606532 | 3.92E-05 | 0.007152316 |
| *Clostridiales bacterium KLE1615* | Disease | 0.021578964 | Disease | 0.116255534 | 2.60E-05 | 0.017530616 |
| *Clostridium sp AF15 49* | Disease | 0.032318349 | Disease | 0.011333499 | 0.000543985 | 0.003264328 |
| *Clostridium sp AF32 12BH* | Disease | 0.057838319 | Disease | 0.062580875 | 0.037016136 | 0.023966635 |
| *Clostridium sp AF34 10BH* | Disease | 0.004566517 | Disease | 0.00146593 | 1.35E-05 | 8.65E-05 |
| *Clostridium sp AM33 3* | Disease | 0.000716203 | Disease | 0.33058676 | 0.028709397 | 0.002213403 |
| *Clostridium sp AM49 4BH* | Disease | 0.000520746 | Disease | 0.066991627 | 7.26E-06 | 0.000392932 |
| *Clostridium sp AT4* | Control | 0.000759788 | Control | 0.173751137 | 0.000678759 | 0.001311243 |
| *Clostridium symbiosum* | Control | 0.000129213 | Control | 0.01645211 | 2.27E-05 | 2.99E-05 |
| *Coprococcus eutactus* | Disease | 0.004587774 | Disease | 0.108717205 | 0.000284984 | 0.0042911 |
| *Dielma fastidiosa* | Control | 0.0221895 | Control | 0.383783063 | 0.04368694 | 0.049101408 |
| *Enterocloster bolteae* | Control | 0.004851373 | Control | 0.665044449 | 0.010022769 | 0.021734162 |
| *Enterocloster citroniae* | Control | 0.004148879 | Control | 0.727794163 | 0.013478376 | 0.020540819 |
| *Enterocloster clostridioformis* | Control | 0.001872156 | Control | 0.402751187 | 0.013752607 | 0.006175444 |
| *Erysipelatoclostridium ramosum* | Control | 1.47E-05 | Control | 0.217310108 | 0.013526699 | 4.37E-05 |
| *Eubacterium ramulus* | Disease | 0.004687929 | Disease | 0.476228466 | 0.000118587 | 0.015861247 |
| *Faecalibacterium prausnitzii* | Disease | 0.002438769 | Disease | 0.042827301 | 0.000779552 | 0.001061885 |
| *Faecalibacterium SGB15346* | Disease | 0.000449727 | Disease | 0.074112768 | 0.000296444 | 0.000376936 |
| *Faecalicatena fissicatena* | Disease | 0.281560706 | Disease | 0.007492168 | 0.000554176 | 0.015106772 |
| *Firmicutes bacterium AF16 15* | Disease | 0.016871189 | Disease | 5.46E-05 | 1.27E-06 | 1.37E-05 |
| *Flavobacteriales bacterium* | Disease | 0.036217295 | Disease | 0.0367147 | 0.010450909 | 0.010136087 |
| *Fusicatenibacter saccharivorans* | Disease | 0.11745915 | Disease | 0.033921004 | 0.000613232 | 0.025999304 |
| *Gemmiger formicilis* | Disease | 0.020214934 | Disease | 0.17433367 | 0.006153692 | 0.023428922 |
| *GGB3277 SGB4327* | Disease | 0.198643459 | Disease | 0.015057441 | 0.003991819 | 0.020375494 |
| *GGB3571 SGB4778* | Disease | 0.094604179 | Disease | 0.02605489 | 0.019008263 | 0.017268122 |
| *GGB55957 SGB77276* | Control | 0.026925648 | Control | 0.158952231 | 0.021881974 | 0.027621684 |
| *GGB9602 SGB15031* | Disease | 0.000601699 | Disease | 0.371571113 | 0.020844004 | 0.002102886 |
| *GGB9619 SGB15067* | Disease | 0.626931564 | Disease | 0.003452727 | 0.017285675 | 0.015445689 |
| *GGB9632 SGB15089* | Disease | 0.106452572 | Disease | 0.010650892 | 0.018957173 | 0.008823537 |
| *GGB9633 SGB15091* | Disease | 0.000260836 | Disease | 0.154413164 | 0.001602224 | 0.000447864 |
| *GGB9646 SGB15123* | Disease | 0.538095466 | Disease | 0.001042805 | 0.004881767 | 0.004761493 |
| *GGB9730 SGB15291* | Disease | 0.03373171 | Disease | 0.039144779 | 0.029114062 | 0.01007455 |
| *GGB9758 SGB15368* | Disease | 0.008076437 | Disease | 0.250514091 | 0.009921428 | 0.014573641 |
| *Hungatella hathewayi* | Control | 0.002357967 | Control | 0.891544223 | 0.02683962 | 0.015061977 |
| *Lachnospira eligens* | Disease | 0.006655218 | Disease | 0.094037174 | 2.09E-05 | 0.00524228 |
| *Lachnospira sp NSJ 43* | Disease | 0.05253975 | Disease | 0.165233005 | 0.001293393 | 0.049887824 |
| *Lachnospiraceae bacterium WCA3 601 WT 6H* | Disease | 1.35E-05 | Disease | 0.002609817 | 7.89E-07 | 6.39E-07 |
| *Longicatena caecimuris* | Control | 0.013850896 | Control | 0.434252244 | 0.047921936 | 0.036771585 |
| *Mediterraneibacter glycyrrhizinilyticus* | Control | 0.00060818 | Control | 0.603384562 | 0.017545355 | 0.003269757 |
| *Oscillibacter sp ER4* | Disease | 5.01E-05 | Disease | 0.108409603 | 0.00387694 | 7.13E-05 |
| *Oxalobacter SGB9385* | Disease | 0.154171031 | Disease | 0.043450673 | 0.014293126 | 0.040232009 |
| *Paraprevotella xylaniphila* | Disease | 0.338880814 | Disease | 0.016616368 | 0.019178562 | 0.034796422 |
| *Roseburia faecis* | Disease | 0.015246648 | Disease | 0.080769176 | 0.000755789 | 0.009481688 |
| *Roseburia hominis* | Disease | 0.005167799 | Disease | 0.008604278 | 5.68E-05 | 0.000490042 |
| *Roseburia intestinalis* | Disease | 4.90E-05 | Disease | 0.557330408 | 0.000155559 | 0.000314402 |
| *Roseburia inulinivorans* | Disease | 0.081581758 | Disease | 0.060529245 | 0.003348298 | 0.031163142 |
| *Roseburia sp AF02 12* | Disease | 0.010049787 | Disease | 0.152695888 | 0.000488085 | 0.011477767 |
| *Ruminococcaceae unclassified SGB4191* | Disease | 0.133702698 | Disease | 0.015754165 | 0.003783223 | 0.015087511 |
| *Ruminococcus gnavus* | Control | 0.000729622 | Control | 0.072080192 | 0.018266992 | 0.000570771 |
| *Ruminococcus lactaris* | Disease | 0.006787285 | Disease | 0.125032398 | 5.39E-05 | 0.006850049 |
| *Ruminococcus sp NSJ 71* | Disease | 0.004001143 | Disease | 0.978775686 | 0.003347636 | 0.025622379 |
| *Senegalimassilia anaerobia* | Disease | 0.007098048 | Disease | 0.150924629 | 0.009908741 | 0.008397591 |
